# Proteolytic cleavage of Beclin 1 exacerbates neurodegeneration

**DOI:** 10.1186/s13024-018-0302-4

**Published:** 2018-12-29

**Authors:** Gregor Bieri, Kurt M. Lucin, Caitlin E. O’Brien, Hui Zhang, Saul A. Villeda, Tony Wyss-Coray

**Affiliations:** 1 Department of Neurology and Neurological Sciences, Stanford University School of Medicine, Stanford, CA 94305 USA; 2 Neurosciences PhD Program, Stanford University School of Medicine, Stanford, CA 94305 USA; 3 Center for Tissue Regeneration, Repair and Restoration, VA Palo Alto Health Care System, 3801 Miranda Avenue, 154W, Palo Alto, CA 94304 USA

**Keywords:** Beclin 1, Neurodegeneration, Alzheimer’s disease, Hippocampus, Caspase, Apoptosis

## Abstract

**Background:**

Neuronal cell loss contributes to the pathology of acute and chronic neurodegenerative diseases, including Alzheimer’s disease (AD). It remains crucial to identify molecular mechanisms sensitizing neurons to various insults and cell death. To date, the multifunctional, autophagy-related protein Beclin 1 has been shown to be both necessary and sufficient for neuronal integrity in neurodegenerative models associated with protein aggregation. Interestingly, besides its role in cellular homeostasis, Beclin 1 has also been ascribed a role in apoptosis. This makes it critical to elucidate whether Beclin 1 regulates neuronal death and survival across neurodegenerative conditions independent of protein clearance. Here, we provide experimental evidence for a direct functional link between proteolytic cleavage of Beclin 1 and apoptotic neuronal cell loss in two independent models of neurodegeneration in vivo.

**Methods:**

Proteolytic cleavage of Beclin 1 was characterized in lysates of human AD brain samples. We developed viral tools allowing for the selective neuronal expression of the various Beclin 1 forms, including Beclin 1 cleavage products as well as a cleavage-resistant form. The effect of these Beclin 1 forms on survival and integrity of neurons was examined in models of acute and chronic neurodegeneration in vitro and in vivo. Markers of neuronal integrity, neurodegeneration and inflammation were further assessed in a Kainic acid-based mouse model of acute excitotoxic neurodegeneration and in a hAPP-transgenic mouse model of AD following perturbation of Beclin 1 in the susceptible CA1 region of the hippocampus.

**Results:**

We find a significant increase in caspase-mediated Beclin 1 cleavage fragments in brain lysates of human AD patients and mimic this phenotype in vivo using both an excitotoxic and hAPP-transgenic mouse model of neurodegeneration. Surprisingly, overexpression of the C-terminal cleavage-fragment exacerbated neurodegeneration in two distinct models of degeneration. Local inhibition of caspase activity ameliorated neurodegeneration after excitotoxic insult and prevented Beclin 1 cleavage. Furthermore, overexpression of a cleavage-resistant form of Beclin 1 in hippocampal neurons conferred neuroprotection against excitotoxic and Amyloid beta-associated insults in vivo.

**Conclusions:**

Together, these findings indicate that the cleavage state of Beclin 1 determines its functional involvement in both neurodegeneration and neuroprotection. Hence, manipulating the cleavage state of Beclin 1 may represent a therapeutic strategy for preventing neuronal cell loss across multiple forms of neurodegeneration.

**Electronic supplementary material:**

The online version of this article (10.1186/s13024-018-0302-4) contains supplementary material, which is available to authorized users.

## Background

Neuronal cell loss is a hallmark of both chronic and acute neurodegenerative diseases such as Alzheimer’s disease (AD) [1, 2] and cerebral ischemia [3]. To date, it remains crucial to identify and understand the molecular mechanisms contributing to neuronal cell death as a potential means to develop broad therapeutic strategies to treat neurodegeneration.

Previous reports have described a direct role for the multifunctional protein Beclin 1 in neurodegeneration [4–8]. These effects have mostly been attributed to its role in autophagy, an intracellular homeostatic degradation pathway that has been implicated in a variety of neurodegenerative diseases [9–11]. However, unlike other autophagy-related proteins, Beclin 1 knock-out mice are embryonic lethal [9, 10] and neuron-specific deletions lead to more severe phenotypes than that of other autophagy-related genes [11–15]. Cumulatively, these findings suggesting a role for Beclin 1 beyond autophagy. Several studies have shown that RIPA-soluble Beclin 1 levels are decreased in the brains of human AD patients [5, 8, 16, 17]. Furthermore, heterozygous Beclin 1^+/−^ hAPP transgenic mice exhibit increased Aβ plaque load and enhanced neurodegenerative changes [5]. Decreased Beclin 1 level and altered distribution have further been reported in poly-glutamine diseases including Huntington’s disease [7, 18]. Conversely, overexpression of Beclin 1 in several models of protein aggregation has been shown to rescue pathology as measured by reduced protein aggregates and associated neuritic degeneration [5, 18–20]. A direct mechanism linking Beclin 1 function to neuronal cell loss or neuronal survival, independent of autophagy and protein clearance, has yet to be explored.

While its function in autophagy is well established, other roles are less widely appreciated in mammalian cells. Beclin 1 is a complex protein with different functional domains involved in a variety of biological processes [21, 22]. The evolutionarily conserved domain and the coiled-coil domain act as a scaffold for several binding partners. Due to its interaction with different protein complexes, Beclin 1 has been shown to regulate a series of additional membrane and organelle trafficking events including endocytosis and receptor recycling [12, 21, 23–25]. The Bcl-2 homology-3 domain (BH3) allows Beclin 1 to interact with the Bcl2-family of proteins, which are known apoptotic damage sensors [26, 27]. Additionally, it has been reported that Beclin 1 contains caspase-cleavage sites [28, 29] and can be cleaved by caspases in vitro [30, 31]. Together, the BH3 domain and caspase-cleavage sites provide a direct mechanistic link to apoptosis and cell death. Studies in various cell lines in vitro have established a link between strong caspase activating stimuli and Beclin 1 cleavage [28, 29, 32]. The presence of the Beclin 1 fragments has further been detected histologically in post-mortem AD forebrain tissue, providing an additional correlative link between cell death and Beclin 1 cleavage [16, 33]. However, the physiological relevance of Beclin 1 cleavage and the functional roles of the resulting cleavage fragments in neurons has yet to be investigated under either normal or disease states in vivo.

Given the potential functional implications of Beclin 1 cleavage, the question arises whether the involvement of Beclin 1 in neurodegenerative diseases extends beyond the loss of its canonical role in autophagy and clearance of protein aggregates. Accordingly, we hypothesized that Beclin 1 cleavage occurs during neurodegeneration, directly contributing to apoptosis-mediated neuronal cell death. We developed virus-based tools to manipulate Beclin 1 cleavage and express the resulting fragments in two mouse models of neurodegeneration. We report that Beclin 1 is cleaved in vivo in an acute Kainic acid-based model of neurodegeneration and demonstrate a functional role for the cleavage fragments in neuronal death. Furthermore, the C-terminal cleavage fragment also exacerbates degenerative phenotypes in a human APP-transgenic mouse model of AD [34]. Besides leading to a loss of its endogenous function, cleavage of Beclin 1 can, in part, exacerbate the neurodegeneration phenotype by creating a fragment that sensitizes neurons to stressors associated with degenerative disease processes. Furthermore, caspase inhibitors and neural overexpression of a caspase-cleavage resistant form of Beclin 1 confers neuroprotection in vivo. Our data demonstrate a dual role for Beclin 1 in both neuronal cell loss and neuroprotection, making it an interesting target for future therapeutic interventions to treat neurodegenerative diseases.

## Materials and methods

### Mice

For all Kainic acid (KA) lesion experiments wild type female FVB/N mice from The Jackson Laboratories were used. FVB/N mice have been reported to be susceptible to KA lesion [35–37]. The mice were 8–10 weeks of age by the beginning of the experiments. The genetic Beclin 1-LacZ reporter mouse line expressed β-galactosidase under the endogenous Beclin 1 locus. T41 hAPP transgenic mice express human APP751 (London V717I and Swedish K670 M/N671 L mutations) under the murine Thy1 promoter [34]. The Beclin 1 reporter mice and hAPP-transgenic mice were bred on a C57bl/6 genetic background. All animal experiments were performed in accordance with institutional guidelines and approved by the local IACUC of the Veterans Administration Palo Alto Health Care System.

### Stereotaxic injections

Animals were placed in a stereotaxic frame and anesthetized with 2% isoflurane (2 L/min oxygen flow rate) delivered through an anesthesia nose cone. Ophthalmic eye ointment was applied to the cornea to prevent desiccation during surgery. The area around the incision was trimmed. Viral or drug solutions were injected bilaterally into the dorsal hippocampi using the following coordinates: (from bregma) anterior = − 2 mm, lateral = 1.5 mm, (from skull surface) depth = − 1.8 mm. Per hemisphere, 1 μl volume was injected stereotaxically at a rate of 100 nl/min using a 5 μl Hamilton syringe. To limit reflux along the injection track, the needle was maintained in situ for five minutes, before being slowly retrieved. The skin was closed using silk suture. Each mouse was injected subcutaneously with the analgesic Buprenorphine, single-housed and monitored during recovery. Beclin 1 viruses (Beclin-AAV) were stereotaxically injected into one hippocampus whereas the control virus (Control-AAV), based on the same viral plasmid, was injected as an internal control into the contralateral hemisphere. The caspase 3 inhibitor Z-DEVD-FMK (R&D Systems) was resuspended following the manufacturer’s instructions in DMSO and PBS to reach a final concentration of 50 μM. DMSO diluted in PBS was used as vehicle control. Adult 6–8 week old mice were stereotaxically injected for Kainic acid experiments. hAPP-transgenic mice were injected at 6 months and sacrificed 3 months later.

### Kainic acid administration

Kainic acid (Tocris Bioscience) was dissolved in sterile PBS and injected subcutaneously into the armpit area of the forelimb (10–50 mg/kg body weight) as previously described [37]. 25 mg/kg body weight was used for all AAV experiments. Animals were observed post injection and seizure activity was reported from 0 to 8; with 0 showing no apparent behavioral changes and 8 representing death [38]. Animals that did not reach at least stage 3 (unilateral forelimb clonus) were excluded from analysis. Animals were examined for 5 days post injection. Body weight was tracked and animals were euthanized if weight loss exceeded 20% bodyweight. Female FVB/N mice were used for KA lesion experiments unless indicated otherwise.

### AD tissue

Brain tissues from confirmed AD and age-matched, non-demented, non-pathological controls were obtained from ADRC at the University of California San Diego, The Institute for Brain Aging and Dementia Tissue Repository at the University of California Irvine, Stanford Brain Bank at Stanford University, and The L.J. Roberts Alzheimer’s Center at Banner Sun Health Research Institute in strict accordance with all ethical and institutional guidelines. Autopsy brain tissue from the frontal cortex of 12 neuropathologically confirmed AD case and 11 non-demented cases diagnosed as normal was studied. Age at death (AD: 79.8 ± 6.1 vs. Controls: 82.6 ± 7.7 years) was not significantly different between the two groups. The AD group had a mean MMSE score of 5 ± 3.4, whereas the control group score was 29.6 ± 0.5.

### Tissue processing

Mice were anesthetized with chloral hydrate (400 mg/kg; Sigma Aldrich) and transcardially perfused with 1x PBS. Brains were removed. For stereotaxic injections experiments both hemispheres were postfixed in phosphate-buffered 4% paraformaldehyde (PFA) pH 7.4, at 4 °C for 48 h. Brains were then stored in 30% sucrose in 1x PBS at 4 °C. For all other experiments only one hemibrain was postfixed in PFA whereas the other hemibrain was snap frozen on dry ice and stored at − 80 °C for biochemical analysis. PFA fixed brains were sectioned at 35 μm (coronal sections) with a freezing microtome (Leica) and stored in cryoprotective medium (30% glycerol, 30% ethylene glycol) at − 20 °C. For stereotaxic injection experiments sections with visible needle tracks were used for further processing and analysis. For biochemical analysis, hippocampi were subdissected, snap frozen in liquid nitrogen and dissociated using a CryoGrinder. RNA and protein extraction was performed separately.

### Viral plasmids

Adeno-associated viral (AAV)-based delivery systems were used for in vivo hippocampal expression of various Beclin 1 and control constructs. Bi-cistronic AAV plasmids were generated, expressing Beclin 1 in frame with a T2A-hrGFP reporter. Beclin 1-T2A was cloned into the AAV backbone using the restriction enzymes ClaI and EcoRI. Lentiviral plasmids were used for in vitro experiments. Beclin 1 was cloned into third-generation lentiviral plasmids with and without an IRES eGFP reporter. Beclin 1 was expressed from Synapsin 1 or CMV promoters. For each viral backbone, four Beclin 1 constructs were generated expressing FL-beclin (nt 1–1350), N-beclin (nt 1–399), C-beclin (nt 444–1350) or a caspase-cleavage resistant CR-beclin. Caspase-cleavage sites were mutated to generate a caspase-resistant form of Beclin 1. Point mutations were introduced using the QuikChange Lightning Site-Directed Mutagenesis Kit (Agilent) to change Aspartic acids D133, D149 to Alanine in the amino acid sequence. All coding sequences were verified by Sanger sequencing and Beclin 1 expression was tested by Western blot analysis.

### Viruses

High-titer AAV were generated at the Stanford Neuroscience Gene Vector and Virus Core. The neuron-specific serotype AAV-DJ was used to efficiently infect neurons over a wide area of the CA1 region of the hippocampus in vivo [39, 40]. AAV titers were between 5 × 10^11^ to 2.2 × 10^12^ infectious viral particles/ml. Equivalent amounts of infectious particles were used for in vivo experiments. Lentiviral vectors were generated using the third generation pRSV-ReV and pMDLg/pRRE packaging plasmids and the pMD2.g VSV-G expressing envelope plasmid (Addgene Cat# 12253, 12251, 12259). HEK293T (ATCC) cells were cultured under standard conditions and used to package lentiviral particles according to standard protocols. Lentivirus-containing medium was harvested after 48 h, centrifuged at 300 g for 5 min to remove cellular debris, and concentrated tenfold with Lenti-X concentrator (Clontech).

### Cell culture experiments

HEK 293 T cells and two neuroblastoma cell lines, B103 cells and Neuro-2a cells (ATCC), were cultured under standard conditions in DMEM media supplemented with 10% fetal bovine serum. For immunocytochemistry and fluorescent microscopy experiments, cells were grown on poly-L-lysine coated coverslips. Cells were plated at 80% confluency for transfection experiments. Lipofectamine 2000 (Invitrogen) was used as transfection reagent. Culture media was exchanged after 6-12 h to reduce toxicity caused by the transfection reagent. For lentiviral transduction, packaged viral particles were directly added to the culture media. The media was changed after 24 h. Gene expression was assessed with the help of fluorescent protein reporters. For apoptosis assays staurosporine (Sigma-Aldrich) was added to the cell culture media at various concentrations (0 to 2 μM). Cells were lysed in RIPA buffer at different time points ranging from 0 to 24 h or fixed with 4%PFA in PBS for 15 min at room temperature.

### Primary neuron and glia cultures

Primary mouse hippocampal and cortical neurons were dissociated into single-cell suspensions from E16.5 mouse brains with a papain dissociation system (Worthington, Cat# LK003153). Neurons were seeded onto poly-L-lysine–coated plates (0.1% (wt/vol)) and grown in Neurobasal medium (Gibco) supplemented with B-27 serum-free supplement (Gibco), GlutaMAX, and penicillin–streptomycin (Gibco) in a humidified incubator at 37 °C, with 5% CO2. Half media changes were performed every 4 or 5 d, or as required. For neurite outgrowth experiments, neurons were transduced with Beclin 1 lentivirus on 1DIV. 30 mM KCl was added on 6DIV. Neurons were fixed with 4% PFA and stained for MAP2. Neurite analysis was performed with ImageJ and NeuronJ software. For survival and autophagic flux experiments, neurons were transduced on 7DIV and analyzed 2–14 days later. For primary glial cultures, brains from P1-P4 pups were dissociated into single-cell suspensions with a papain dissociation system. Cells were seeded onto uncoated tissue culture plates and grown in DMEM with 10% FBS and penicillin–streptomycin .

### Cytotoxicity assays

Cytotoxicity on primary-neuron culture media was measured by LDH release assays (Promega, G1780). Additional cytotoxicity assays were performed with the Caspase-Glo 3/7 Assay (Promega G8090), a luminescence-based readout of caspase 3/7 activity (DEVDase activity), and the CellTiter-Glo Luminescent Cell Viability Assay (G7570), according to the manufacturer’s instructions. Neuronal survival was further quantified using the neuronal marker NeuN. To quantify the numbers of neurons, cells were fixed, stained for the marker NeuN and quantified with ImageJ. Briefly, thresholds were applied to image stacks to detect NeuN+ neuronal nuclei, which were then automatically counted with built-in ImageJ plugins. All images were acquired in a blinded and randomized way.

### Autophagic flux

To measure and visualize autophagic flux, the LC3-GFP reporter construct was cloned into a third generation lentiviral vector. Neuro-2a cells (N2a) were transduced and stable cell lines expressing low level of LC3-GPF were generated. The reporter cell line was transduced with the different Beclin 1 constructs and LC3-GFP puncta were quantified using MetaXpress Software. Rapamycin (10 nM) was used as a positive control for autophagy induction. Bafilomycin A (40 nM) was added to the culture media to block autophagic flux. For LC3II experiments in neurons, primary cells were transduced on 7DIV and analyzed 7 days later. Neurons were treated with Bafilomycin A for 3 h before cell lysis and analysis by Western blot.

### Western bolt analysis

Mice were perfused with PBS, hippocampi were immediately dissected, snap frozen and stored at − 80 °C. Human cortical mid-frontal gray matter tissue was isolated from frozen brain tissue blocks. In vitro, cell lines and primary cells were washes with PBS prior to lysis. Cells or tissue were lysed on ice in 1x RIPA lysis buffer supplemented with Halt protease and phosphatase Inhibitor Cocktail (Roche). Crude lysates were centrifuged at 12,000 g for 10 min at 4 °C to remove cellular debris. Clarified lysates were quantified with a Pierce BCA protein assay. Cell lysates were mixed with 4x NuPage LDS loading buffer, loaded into pre-cast 4–12% Bis-Tris Protein Gels (Thermo Fisher Scientific) and subsequently transferred onto a PVDF or nitrocellulose membrane. The blots were blocked in 5% milk in Tris-Buffered Saline with 0.1% Tween (TBST) and incubated with primary antibodies at 4 °C overnight: anti-Actin (1:10000, Millipore cat# MAB1501), anti-C-terminal Beclin 1 (1:1000, BD Biosciences, Cat# 612112), anti-N-terminal Beclin 1 (1:1000, AnaSpec Cat# 54229), anti-LC3 (1:1000, Cell Signaling Technology Cat# 3868 and 12,741), anti-cleaved-caspase 3 (1:300, Cell Signaling Technology, Cat# 9661 and 9664), anti-NSE (1:10000, Lab Vision Corporation/Thermo Fisher Scientific), anti-Synaptophysin 1 (1:3000, Synaptic Systems Cat# 101002), anti-PSD95 (1:3000, Abcam Cat# ab18258), anti-hrGFP (1:1000, Agilent Technologies, Cat# 240142). Horseradish peroxidase-conjugated secondary antibodies and an ECL kit (GE Healthcare/Amersham Pharmacia Biotech) were used to detect protein signals. Multiple exposures were taken to select images within the dynamic range of the film (GE Healthcare Amersham HyperfilmTM ECL). Protein bands were quantified using ImageJ software/FIJI. Actin bands were used for normalization.

### RNA isolation and quantitative RT-PCR analysis

RNA was isolated from brain tissue and cell pellets using TRIZol reagent (Thermo Fisher Scientific, Cat# 15596026) and PureLink™ RNA Mini Kit following the manufacturer’s instructions. The RNA concentration was determined via Nanodrop and RNA was reverse transcribed using the High-Capacity cDNA Reverse Transcription Kit (Thermo Fisher Scientific, Cat# 4368813). Real time PCR was performed on a Applied Biosystems StepOnePlus Real-Time PCR instrument using 2x TaqMan Universal Master Mix (Cat # 4440040) and gene-specific TaqMan probes against Becn1 (Mm01265461_m1, Hs01007018_m1) and ActB (Mm02619580_g1). ActB was used for normalization. Each sample and primer set was run in triplicates and relative expression level were calculated using the ΔΔCT method.

### β-Galactosidase activity assay

Beclin 1 reporter mice and WT littermate controls were perfused with 1x PBS. Brains were dissected and fixed in 4% PFA for 2 h at 4 °C. Brains were sectioned at 110 μm using a vibratome (Leica). Sections were washed 3 times with 1x PBS (pH 7.2) and incubated in staining solution (5 mM K_3_Fe(CN)_6_, 5 mM K_4_Fe(CN)_6_, 2 mM MgCl_2_, 1 mg/ml X-gal in PBS, pH 7.2) at 37° until tissue was stained blue. Stained tissue was post-fixed in 4% PFA for 24 h and stored in 30% Sucrose in 1x PBS. Brain sections were mounted on Superfrost plus slides (Fisher Scientific), air-dried and coverslipped with entellan (Electron Microscopy Sciences).

### Cresyl violet staining

Brain sections were mounted on Superfrost plus slides (Fisher Scientific), air-dried, stained with 0.02% Cresyl Violet (Sigma-Aldrich) in acetate buffer (pH 3.6), then dehydrated through a series of alcohols, cleared with CitroSolv (FisherBrand) and coverslipped with entellan (Electron Microscopy Sciences). Neuronal loss was assessed based on the appearance/thickness of the Nissl substance in the CA1 pyrimidal cell layer of the hippocampus. 4–5 brain sections were analyzed per animal. Metamorph image software (Molecular Devices, Version 7.6) was used to quantify the percent area occupied in a frame placed over the CA1 area of the hippocampus. All images were acquired and quantified in a blinded fashion.

### Immunohistochemistry

Immunohistochemistry was performed on free-floating sections following standard procedures. For each staining, a total of 4–5 hippocampal brain sections per mouse were used. Sections were pre-treated with 0.6% H_2_O_2_ and 0.1% Triton® X-100 (Sigma-Aldrich) and blocked in 10% of the serum the secondary antibody was raised in. After overnight primary antibody incubation, the staining was revealed using biotinylated secondary antibodies, ABC kit (Vector Laboratories) and 3,3′-diaminobenzidine tetrahydrochloride (DAB, Sigma-Aldrich). Fluorescently labeled secondary antibodies were used for fluorescent and confocal microscopy. Primary antibodies against Calbindin (1:15000, Swant, Cat# 300), CD68 (1:1000, AbD Serotec, Cat# MCA1957GA), cleaved-caspase 3 (1:500, Cell Signaling Technology, Cat# 9661 and 9664), hrGFP (1:10000, Agilent Technologies, Cat# 240142) and NeuN (1:1000, Millipore, Cat# MAB377) were used. A biotinylated anti-Aβ primary antibody (3D6) was used for Amyloid beta plaque detection, as previously described [41]. The immunoreactivity was quantified as percent area covered by the staining, using Metamorph software (Molecular Devices, Version 7.6). All images were acquired and quantified in a blinded fashion.

### Immunocytochemistry

Cells were grown on poly-L-lysine–coated glass coverslips (0.1% (wt/vol) in standard multiwell cell culture plates and were stained through standard immunocytochemistry techniques. Briefly, cells were fixed with 4% PFA, permeabilized with 0.1% Triton X-100, blocked with 5% normal goat serum, and stained with the following antibodies: MAP2 (1:1000, Synaptic Systems, Cat# 188004), NeuN (1:1000, Millipore, Cat# MAB377), cleaved-caspase 3 (1:500, Cell Signaling Technology, Cat# 9661 and 9664). Coverslips were mounted with Prolong Diamond Antifade Mountant with DAPI (Thermo Fisher Scientific). Images were acquired with a Leica DMI6000B inverted fluorescence. All images were acquired and quantified in a blinded fashion.

### Data and statistical analysis

Data are presented as mean + SEM. Statistical analyses were performed with GraphPad Prism software (Version 6.0). Differences between treatment conditions were established using a unpaired Student’s t test (for two conditions). For experiments with > 2 groups, a one-way ANOVA with a Tukey’s post test for multiple comparisons was performed. Pearson r was calculated for correlation analysis. *p* < 0.05 was considered statistically significant. Statistic details are indicated in the respective figure legends.

## Results

### Beclin 1 cleavage in AD brains

**Fig. 1:**
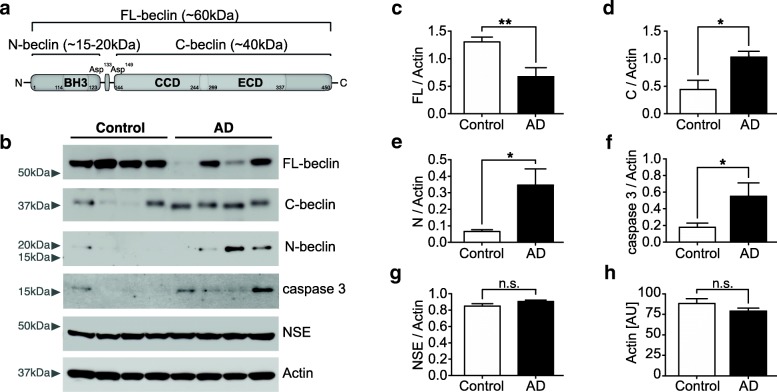
Beclin 1 is cleaved in human Alzheimer’s disease brains. **a** Schematic depicting different domains of Beclin 1 and estimated fragment sizes of cleavage products. Bcl-2-homology 3 domain (BH3), coiled-coil domain (CCD) and evolutionarily conserved domain (ECD). Beclin 1 has two conserved caspase-cleavage sites near the N-terminus. **b** Representative Western blots of RIPA-soluble brain lysates from human Alzheimer’s disease (AD) patients and age-matched controls were probed with anti-Beclin 1, anti-caspase 3, anti-Neuron specific enolase (NSE) and anti-Actin antibodies. **c**-**e** Quantification of FL-beclin (**c**), C-beclin (**d**) and N-beclin (**e**). **f** Quantification of activated cleaved caspase 3. **g**, **h** Quantifications of NSE (**g**) and Actin (**h**), used as loading controls. For all quantitative analyses Beclin 1 and caspase levels were normalized to the actin loading control (*n* = 12 for AD, *n* = 11 for control group). Data expressed as mean + SEM; *p < 0.05; **p < 0.01; unpaired Student’s t-test

To determine whether Beclin 1 cleavage occurs in the context of a neurodegenerative disease we performed Western blot analysis on the RIPA-soluble fraction of cortical brain lysates from AD patients and healthy controls. Consistent with previous findings [5, 16, 17], we observed that full-length Beclin 1 (FL-beclin) levels are decreased in human AD patients (Fig. 1b, c). We hypothesized that the decrease in Beclin 1 may be due to increased degradation or cleavage. Subsequently, using Western blot analysis we also detected a significant increase in both the N-terminal (N-beclin) and C-terminal Beclin 1 fragments (C-beclin) in brain lysates of human AD patients (Fig. 1b, d and e). It has been shown that Beclin 1 has caspase-cleavage sites, and caspase 3 is able to cleave Beclin 1 in a cell free system [29–31]. Therefore, we tested the same Western blots for activated caspase 3 and were able to detect it at low levels (Fig. 1b), observing higher expression in AD brain lysates compared to controls (Fig. 1f). These data suggest higher overall apoptotic activity, possibly contributing to neuronal cell loss. Actin and neuron-specific enolase (NSE) were used as loading controls (Fig. 1g, h). Together these findings show that caspase activity is increased in human AD brains, and furthermore that Beclin 1 is cleaved, potentially accounting in part for the decreased levels of FL-beclin observed in AD.

### Beclin 1 is highly expressed in the CA1 region of the hippocampus

To begin to understand the role of Beclin 1 cleavage in neurodegeneration, we first sought to characterize the expression profile of Beclin 1 in the adult brain using a transgenic mouse model. We generated novel reporter mice that express LacZ under the endogenous locus of the Beclin 1 gene. β-galactosidase activity assays reveal the highest expression of the gene in the pyramidal cell layer of the CA1 and CA3 regions of the hippocampus (Additional file 1: Figure S1a, b). We further confirm expression of Beclin 1 mRNA in the hippocampus and in primary hippocampal neurons and glial cells (Additional file 1: Figure S1c). Although Beclin 1 is expressed at basic levels in every cell in the brain [42, 43], our data show that the expression is enriched in neuronal layers within the hippocampus, indicating a potentially important role for this protein in neurons.

### Beclin 1 is cleaved in neuronal cells in vitro

**Fig. 2:**
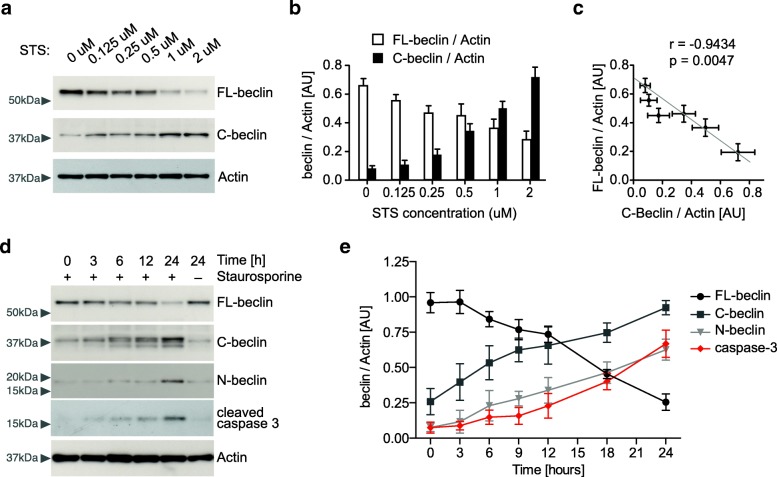
Caspase-mediated proteolytic cleavage of Beclin 1 in neuronal cells. Staurosporine (STS), a known activator of caspase 3, leads to Beclin 1 cleavage and induces apoptosis in the B103 neuronal cell line. **a** Representative Western blot of neuronal cells treated with increasing doses of Staurosporine, probed with anti-C-terminal Beclin 1 and anti-actin antibodies. Cells were lysed 24 h after addition of Staurosporine to the culture media. Arrowheads indicate molecular weight markers (in kDa). **b**, **c** Quantification (**b**) and anti-correlation of FL-beclin with C-beclin level (**c**). **d** Representative Western blot of neuronal cells treated with 1 μM Staurosporine, probed with anti-Beclin 1, anti-cleaved caspase 3 and anti-actin antibodies. Cell were lysed 0 to 24 h after addition of Staurosporine to the culture media. **e** Quantification of Beclin 1 level and cleaved caspase 3 (*n* = 3–4 per time point). Beclin 1 cleavage fragments increase with the activation of caspase 3. Pearson’s correlation, *p < 0.05

Based on in vivo expression patterns, we next wanted to determine if Beclin 1 cleavage occurs in neuronal cells. For this we used the B103 neuronal cell line in combination with the well-known apoptosis inducer Staurosporine [31]. We detected activation of caspase 3 and Beclin 1 cleavage in a dose and time dependent manner (Fig. 2). Specifically, the level of FL-beclin decreased as the level of C-beclin increases (Fig. 2b, c, e). Additionally, Beclin 1 cleavage was paralleled by caspase activation (Fig. 2d, e). Together these data show that Beclin 1 can be cleaved in neuronal cell lines upon the activation of caspases.

### Beclin 1 is cleaved in the adult hippocampus during neurodegeneration

**Fig. 3:**
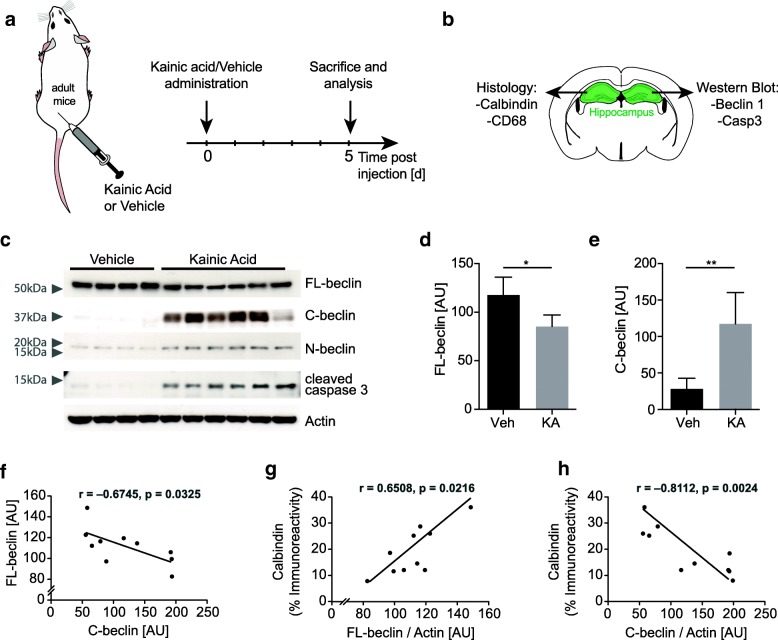
Beclin 1 cleavage, caspase 3 activation and neurodegeneration in a Kainic acid lesion mouse model. **a**, **b** Schematic of experimental design and time line of Kainic acid administration paradigm in mice. Adult mice (*n* = 10 mice per group/dose) were subcutaneously injected with Kainic acid (KA) or PBS and sacrificed five days later. One hemi brain was PFA-fixed for immunohistochemical analysis of neuronal and neuroinflammation markers, while the corresponding contralateral hippocampus was used for Western blot analysis. **c** Representative Western blot of Kainic acid and vehicle treated animals, probed with anti-C-terminal Beclin 1, anti-N-terminal Beclin 1, anti-cleaved-caspase 3 and anti-actin antibodies. Beclin 1 cleavage fragments are elevated in hippocampal lysates of Kainic acid treated animals. **d**, **e** Quantification of FL-beclin and C-beclin (n = 10 mice/group). Data expressed as mean + SEM; *p < 0.05; **p < 0.01; unpaired Student’s t-test. **f** Anti-correlation of FL-beclin and C-beclin. The amount of Beclin 1 fragments increase while FL-beclin 1 level decrease. **g**, **h** Correlation of FL-beclin 1 level (**g**) and C-Beclin 1 level (**h**) with the neuronal marker Calbindin. Beclin 1 was assessed by Western blot, Calbindin was measured by immunohistochemistry. Pearson’s correlation, *p < 0.05

To begin to investigate the functional role of Beclin 1 cleavage in the context of a neurodegenerative disease we sought to utilize existing mouse models exhibiting robust neuronal cell loss. While hAPP transgenic mouse models of AD show reliable Aβ plaque deposition and synaptic and dendritic degeneration, one key hallmark that is not reliably recapitulated is neuronal cell loss and apoptosis [44–46]. In order to first investigate the role of Beclin 1 cleavage in neuronal cell death independent of protein aggregation we used an acute model of neurodegeneration - the Kainic acid (KA) lesion model (Fig. 3a). This model is used to study excitotoxic neurodegeneration and neuronal cell death [37, 47, 48]. Importantly, the KA lesion model is clinically relevant as excitotoxicity is an underlining cell death mechanism in several acute and chronic neurodegenerative diseases [49–52].

Peripheral administration of KA (20–25 mg/kg) has been reported to induce neurodegeneration in the adult hippocampus [48]. Importantly, the hippocampal CA1 and CA3 regions show the highest susceptibility (Additional file 2: Figure S2a-d) [37, 47], coinciding with high expression of Beclin 1 in the brain (Additional file 1: Figure S1a). To quantify neuronal cell loss, we conducted immunohistochemistry analysis on brain tissue of animals administered with KA or vehicle control (Fig. 3b and Additional file 2: Figure S2a-d). Calbindin and Cresyl violet (Nissl staining) were used to assess neuronal integrity and CD68 was used as an indicator of microglial activation. While neuronal markers strongly decreased in KA treated compared to vehicle treated mice, microglial activation increased (Additional file 2: Figure S2a-d). Using Western blot analysis, we also observed caspase 3 activation and Beclin 1 cleavage in KA treated animals (Fig. 3c-f and Additional file 2: Figure S2e, f). Overall, the decrease in FL-beclin protein level lead to a corresponding increase in Beclin 1 fragments (Fig. 3c, f). The decrease in FL-beclin level positively correlated with Calbindin staining and negatively correlated with CD68 staining (Fig. 3g and Additional file 2: Figure S2g). Conversely, C-beclin negatively correlated with Calbindin staining and positively correlated with CD68 staining (Fig. 3h and Additional file 2: Figure S2h). These data show that Beclin 1 is cleaved in vivo in a model of acute neuronal injury, and that Beclin 1 cleavage correlates with neuronal cell loss and microglial activation.

### In vivo viral delivery of Beclin 1 into the adult hippocampus

**Fig. 4:**
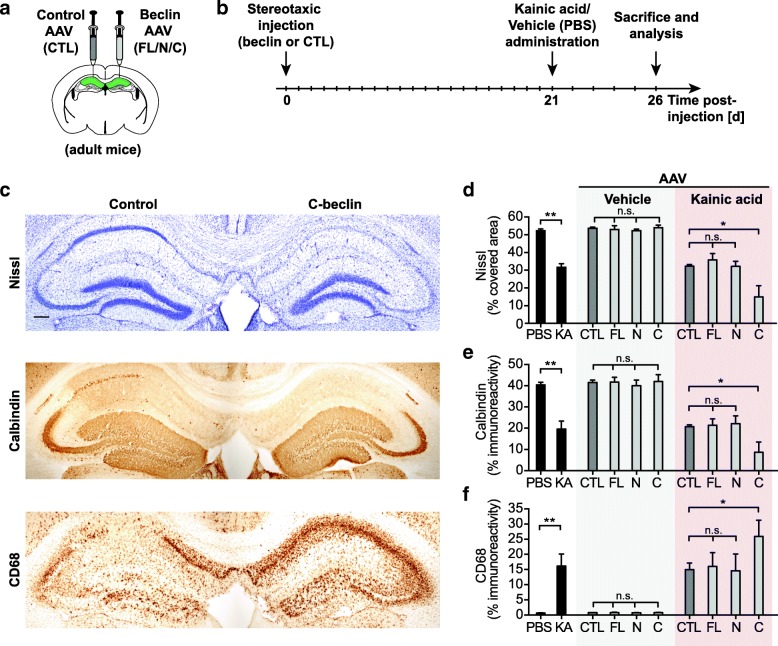
Beclin 1 cleavage fragments exacerbate neurodegenerative phenotypes in vivo. **a**, **b** Schematic representation of the experimental procedures and experimental time line. **a** AAV-mediated stereotaxic delivery of Beclin 1 into the CA1 region of the hippocampus and AAV-control into the contralateral hemisphere. A neuronal AAV serotype was used to selectively express Beclin 1 or control in neurons. **b** Animals were treated with Kainic acid or vehicle after recovery from stereotaxic surgery. **c** Neuronal injury after Kainic acid treatment was assessed by Nissl staining (top panel) and immunohistochemical detection of the neuronal marker Calbindin (middle panel). Microglial activation was assessed by CD68 immunostaining (lower panel). Representative hippocampal images from adjacent sections of one animal expressing C-beclin fragments (right hemisphere) or control (left hemisphere). Neuronal expression of C-beclin exacerbates the degenerative phenotypes of the Kainic acid lesion model. Scale bar 200 μm. **d**-**f** Quantification of Nissl staining (**d**), Calbindin immunostaining (**e**) and CD68 immunostaining (**f**) expressed as percentage area covered by staining in the CA1 region of the hippocampus. Naive Kainic acid and PBS treated animals that did not undergo stereotaxic surgery are included as a reference. C-beclin expression exacerbates neurodegeneration in response to Kainic acid treatment (*n* = 10 mice/group for naive animals; *n* = 5–6 mice/group for AAV-Vehicle condition; *n* = 8–20 mice/group for AAV – Kainic acid condition; 4–5 hippocampal sections / mouse brain). Data expressed as mean + SEM; *p < 0.05; **p < 0.01 compared by unpaired Student’s t-test or one-way ANOVA with a Tukey’s post test for multiple comparisons

In order to investigate the role of the two Beclin 1 cleavage fragments independently we generated novel adeno-associated viruses (AAV) that allow us to selectively overexpress different cleavage forms of Beclin 1 in CA1 neurons in vivo. The bicistronic constructs express either the N or C-terminal cleavage fragments and a GFP reporter (Additional file 3: Figure S3). Additionally, a FL and a caspase-resistant form (CR-beclin) with mutated caspase-cleavage sites were generated based on previously identified caspase cleavage sites [29]. An empty GFP-only plasmid was used as a control (Additional file 3: Figure S3b, c). Plasmids were tested for Beclin 1 and GFP expression in neuronal cell lines (Additional file 3: Figure S3d). Subsequently, we characterized Beclin 1 overexpression in vivo by locally delivering virus stereotaxically into the hippocampus of adult mice (Fig. 4a, Additional file 4: Figure S4 and Additional file 5: Figure S5). All AAVs that were generated showed comparable infection rates and could not be detected on the contralateral side as determined by GFP expression (Additional file 4: Figure S4a and Additional file 5: Figure S5). All viruses showed a similar distribution in the CA1 area and a high specificity for neurons as determined by double-staining of GFP-positive infected cells with the neuronal marker NeuN (Additional file 4: Figure S4b, c and Additional file 5: Figure S5a-c). Quantification showed over 95% of GFP-positive cells expressing NeuN in the CA1 area of the hippocampus. Our data show that we are able to overexpress the different Beclin 1 cleavage fragments with high spatio-temporal control in hippocampal neurons in the CA1 region.

### Beclin 1 cleavage fragments exacerbate neurodegeneration in vivo

Using AAV-mediated delivery into the hippocampus we overexpressed either the C or N-terminal cleavage fragments into the hippocampus of one hemisphere, while the control virus was injected into the hippocampus of the contralateral side serving as an internal control (Fig. 4a). After recovery from viral delivery, animals were lesioned with KA or vehicle control by subcutaneuous injection (Fig. 4b). Brains were collected and analyzed after 5 days. In the absence of any excitotoxic insult, overexpression of Fl-beclin, C-beclin or N-beclin did not lead to neuronal cell loss or changes in microglia activation as compared to a control virus or uninjected animals (Additional file 4: Figure S4d-g). This shows that viral delivery and overexpression of Beclin 1 fragments alone does not have any neurotoxic effects. Surprisingly, if excitotoxicity was induced using the KA injection paradigm, overexpressing C-beclin significantly exacerbated the neurodegeneration phenotype as assessed by Nissl, Calbindin and CD68 staining (Fig. 4c-f). In contrast, overexpressing N-beclin did not alter the response to the excitotoxic insult (Fig. 4d-f). Further, expression of a control virus (CTL) or the stereotaxic surgery alone did not lead to any detectable difference from uninjected KA-treated animals (Fig. 4d-f and Additional file 6: Figure S6).

Previously we found that overexpressing Beclin 1 in a model of AD reduced disease-associated amyloid plaques [5]. Similar findings have also been reported for other models of protein aggregation diseases [7, 18–20]. These protective effects were generally associated with increased autophagy, decreased protein aggregation burden and decreased amyloidosis-associated neurodegeneration. However, using these models it is difficult to determine whether increasing Beclin 1 level contributes most to neuroprotection via autophagic clearing of protein aggregates or via other intrinsic neuronal survival mechanisms. Surprisingly, little data is available that elucidates the function of Beclin 1 in neurodegenerative models in the absence of protein aggregation. Therefore, we used the in vivo AAV approach described above for the cleavage fragments and overexpressed FL-beclin in the hippocampus prior to KA lesion (Fig. 4a, b). However, unlike in protein aggregation models, FL-beclin overexpression did not rescue the neurodegeneration phenotype in the KA excitotoxic cell death model (Fig. 4d-f). Consistent with our findings for N and C-beclin, overexpressing FL-beclin elicited no changes in neurodegeneration or microglia activation in the vehicle treated group (Fig. 4d-f).

Together these data show that the C-terminal cleavage fragment exacerbates neuronal cell loss in an excitotoxic neurodegeneration model that lacks protein aggregates, whereas the N-terminal fragment does not have a functional role in this paradigm. Interestingly, overexpressing C-beclin alone in the absence of excitotoxic injury is not sufficient to induce any detectable degenerative phenotypes in neurons in vivo (Fig. 4c-f). Furthermore, overexpressing FL-beclin does not alter neuronal survival with excitotoxic injury, indicating that our observed effects on neuronal cell loss are independent of those previously observed in protein-aggregation disease models [5, 18–20]. Because the C-beclin fragment only has robust functional consequences with excitotoxic insult, we speculated that it may be involved in sensitizing neurons to cell death. In line with this finding, a positive feedback loop on apoptosis has previously been suggested in cell line experiments in vitro [28].

### Caspase 3 inhibitor DEVD rescues neurodegeneration in vivo

**Fig. 5:**
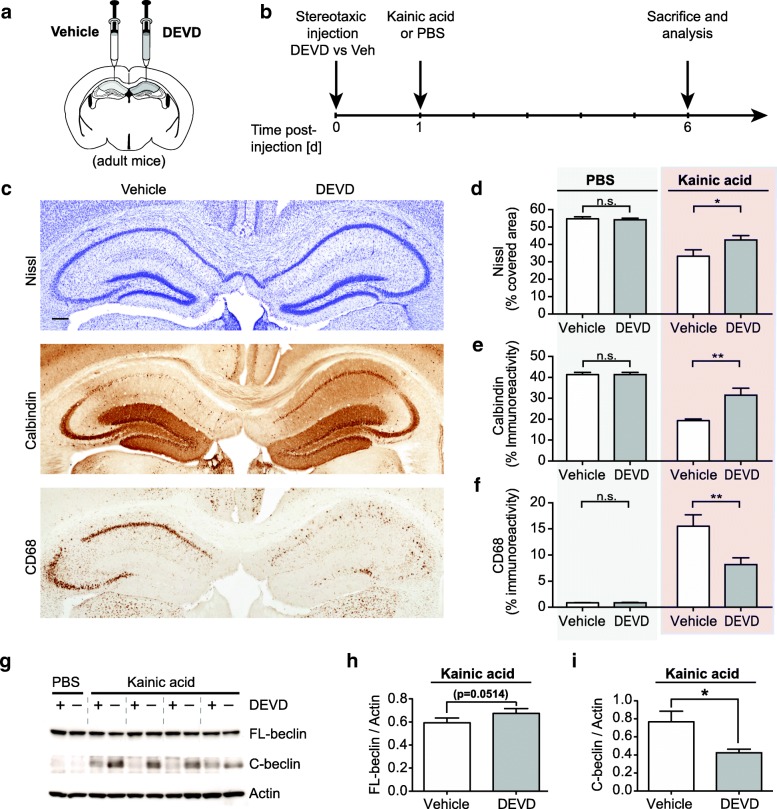
Inhibition of caspase 3 is neuroprotective in the Kainic acid lesion model. **a**, **b** Schematic representation of the experimental procedure and experimental time line. **a** Local stereotaxic delivery of the caspase 3 inhibitor z-DEVD-FMK (DEVD) or vehicle control into the CA1 area of the hippocampus. **b** Adult mice were lesioned with Kainic acid one day post stereotaxic injection with DEVD or vehicle control. **c** Neuronal injury was assessed by Nissl staining (top panel) and Calbindin immunostaining (middle panel). Microglial activation was assessed by CD68 immunostaining (lower panel). Representative hippocampal images from adjacent section of one animal are shown (scale bar: 200 μm). Local pretreatment with the caspase 3 inhibitor DEVD reduces the neurodegeneration phenotype observed in the Kainic acid lesion model compared to vehicle control. **d-f** Quantification of Nissl staining (**d**), Calbindin immunostaining (**e**) and CD68 immunostaining (**f**) expressed as percentage area covered by the staining in the CA1 region of the hippocampus. (*n* = 6–9 mice per group, 4–5 sections per mouse brain). **g** Representative Western blot of hippocampal lysates of mice pretreated with DEVD or vehicle control prior to Kainic acid lesion or PBS injection. Membranes were probed with anti-C-beclin and anti-Actin antibodies. Individual animals separated by dashed line. **h**, **i** Quantification of FL-beclin (**h**) and C-beclin (**i**) (Western blot analysis: *n* = 5 mice per group, lysates from DEVD and vehicle control hemisphere analyzed for each animal). Data expressed as mean + SEM; *p < 0.05; **p < 0.01; compared by paired Student’s t-test

A hallmark of apoptosis is the activation of caspases, including the executioner/effector caspase 3. Caspases can cleave a wide range of substrates during apoptosis that eventually lead to cell death [53–57]. Beclin 1 has also previously been shown to be cleaved by caspases in a cell free system and in cell lines in vitro [28, 30]. We hypothesized that overexpressing FL-beclin did not elicit beneficial effects in our model of neurodegeneration because the protein itself is still subject to caspase cleavage, which may consequently lead to a loss of its function while also generating more C-terminal fragments. We therefore reasoned that abrogating caspase activation could in turn block cleavage of various substrates, including Beclin 1, and promote neuronal survival. To examine this possibility, caspase 3 specific inhibitor Z-DEVD-FMK (DEVD) or vehicle control were stereotaxically injected into contralateral sides of the hippocampus prior to the peripheral administration of KA or PBS control (Fig. 5a, b). DEVD treatment rescued the neuronal cell loss, underlining the role of caspase activation and apoptosis in this model of neurodegeneration. Specifically, in the side treated with the caspase inhibitor both the neuronal marker Calbindin and Nissl staining significantly increased, while less microglial activation was observed in the CA1 region of the hippocampus (Fig. 5c-f). Similarly, using Western blot analysis we detected a trend towards increased FL-beclin level in the hemisphere that was treated with DEVD, whereas more of the C-beclin was found in the vehicle side (Fig. 5g-i). These data show that caspase activation and cleavage of caspase substrates, including Beclin 1, contribute to neuronal cell loss in the KA lesion model. Correspondingly, treating the hippocampus locally with the caspase 3 inhibitor DEVD is neuroprotective and prevents Beclin 1 cleavage in vivo.

### Expression of caspase-resistant Beclin 1 rescues neurodegeneration

**Fig. 6:**
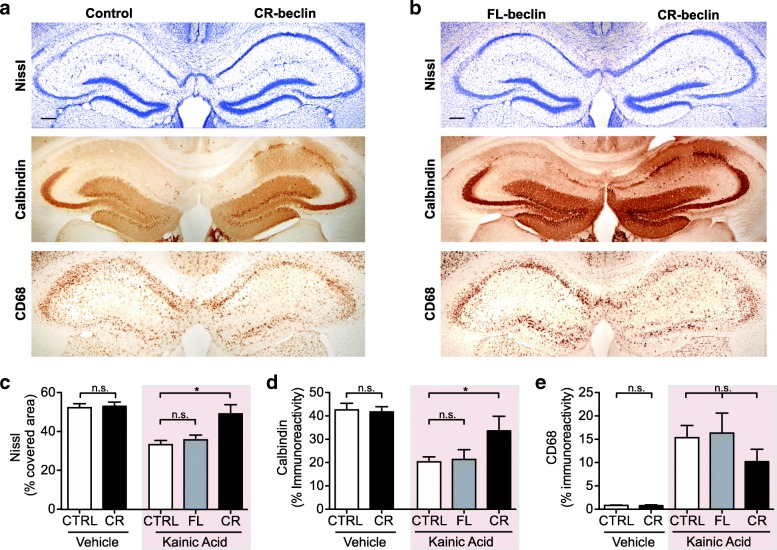
Caspase-resistant Beclin 1 rescues neurodegeneration in vivo. **a**, **b** Adult mice were lesioned with Kainic acid three weeks post stereotaxic delivery of wild-type full length (FL) or caspase-resistant (CR)-beclin-AAV into the hippocampus and Control-AAV into the contralateral hemisphere. **a** Comparison of neuronal CR-Beclin 1 to control-AAV expression. Neuronal injury was assessed by Nissl staining (top panel) and Calbindin immunostaining (middle panel). Microglial activation was assessed by CD68 immunostaining (lower panel). Representative hippocampal images from adjacent sections of one animal expressing CR-Beclin (right hemisphere) and control (left hemisphere) (scale bar 200 μm). **b** Direct comparison of FL-Beclin 1 to CR-Beclin 1 in Kainic acid lesioned mice. FL-beclin-AAV and CR-beclin-AAV were stereotaxically injected into contralateral hippocampi of the same animal. Neuronal injury was assessed by Nissls staining (top panel) and Calbindin immunostaining (middle panel). Microglial activation was assessed using CD68 immunostaining (lower panel). Representative hippocampal images from adjacent sections (scale bar: 200 μm). **c**-**e** Quantification of Nissl staining (**c**), Calbindin immunostaining (**d**) and CD68 immunostaining (**e**) expressed as percentage area covered by staining in the CA1 region of the hippocampus (*n* = 8–12 mice/group; 4–5 sections per mouse brain). Data expressed as mean + SEM; *p < 0.05; compared by unpaired Student’s t-test and one-way ANOVA with a Tukey’s post test for multiple comparisons

Caspase 3 cleaves many substrates, including various autophagy-related genes, of which any number of them may be responsible for the neuroprotective effect observed upon DEVD administration in our neurodegeneration model [31]. To specifically test the contribution of Beclin 1 cleavage, we overexpressed a caspase-resistant form of Beclin 1 (CR-beclin) in hippocampal neurons in vivo using the AAV-mediated approach described above (Fig. 4a, b). Consistent with previous reports, point mutations were introduced in the cDNA sequence of Beclin 1 to render it resistant to caspase cleavage [29]. Excitingly, under excitotoxic conditions, CR-beclin overexpression significantly increased neuronal survival resulting in higher Calbindin expression and Nissl staining compared to the control AAV side (Fig. 6a). To directly compare FL-beclin to CR-beclin, we stereotaxically injected virus encoding either FL-beclin or CR-beclin into contralateral sides of the hippocampus (Fig. 6b). The hemisphere overexpressing CR-beclin showed significantly reduced neuronal cell loss in the pyramidal cell layer of the hippocampus compared to the contralateral cleavable FL-beclin side, as assessed by Calbindin and Nissl staining (Fig. 6c, d). No significant difference in microglial activation was detected (Fig. 6e). Importantly, expression levels of FL-beclin and CR-beclin in the hippocampus were comparable between the two groups (Additional file 5: Figure S5d-g). Additionally, no neurotoxic effects were observed in vehicle treated animals in the absence of KA (Fig. 6c, d and Additional file 5: Figure S5h, i).

Together these experiments demonstrate that specifically CR-beclin, but not cleavable full-length Beclin 1, rescues neurodegeneration in a KA lesion model, highlighting the functional relevance of Beclin 1 cleavage in vivo.

### Beclin 1 cleavage modulates pathology in a hAPP-transgenic model of AD

Protein aggregation is a key histopathological hallmark of many aging-associated neurodegenerative diseases. In AD, Amyloid beta (Aβ) pathology is, at least in part, driving degenerative processes leading to neuronal cells loss. Thus, we sought to apply the previously described AAV-mediated Beclin 1 manipulations to a hAPP transgenic mouse model of AD. Specifically, we examined whether expressing CR-beclin and C-beclin in a hAPP mouse model would have protective and sensitizing effects consistent with those observed in the KA model. We expressed the different Beclin 1 forms in the CA1 region of the hippocampus at the onset of Aβ pathology in 6 months old mice and aged them for 3 months (Additional file 7: Figure S7a). Beclin 1 AAV was delivered into the CA1, whereas the control virus was injected at the same location into the contralateral hemisphere. After 3 months, we observed that FL-beclin overexpression reduced amyloid plaques and associated degenerative and inflammatory phenotypes (Additional file 7: Figure S7b, c), in line with our previous findings using lentiviral Beclin 1 overexpression [5]. Importantly, Aβ pathology was selectively reduced in the CA1 area of the hippocampus, while no changes were observed in the dentate gyrus (DG) which was not targeted by the viral injection (Additional file 7: Figure S7c, d). Additionally, CR-beclin expression also rescued Aβ burden and associated degenerative phenotypes to a similar extent (Additional file 7: Figure S7e, f).

Since we previously observed an increase in Beclin 1 cleavage in brain lysates from human AD subjects (Fig. 1) [5], similar to the KA lesion model (Fig. 3), we next sought to investigate whether the resulting fragments would also have a sensitizing effect in this more chronic hAPP mouse model of neurodegeneration. Neither C-beclin nor N-beclin altered CA1 Aβ plaque burden when expressed in hAPP mice (Additional file 8: Figure S8a, b, e, f). However, C-beclin expression exacerbated the neurodegenerative and inflammatory phenotype as assessed by Calbindin and CD68 immunostaining in the CA1 area of the hippocampus (Additional file 8: Figure S8a-d). In contrast, expressing N-beclin did not alter the degenerative response to Aβ pathology in hAPP mice (Additional file 8: Figure S8e-h). Importantly, we did not observe any degeneration when C-beclin or N-beclin were expressed in WT brains (Additional file 4: Figure S4d-g). These data suggest that Aβ pathology is sufficient for C-beclin to sensitize neurons to degeneration.

Cumulatively, these data indicate that maintaining elevated levels of Beclin 1 is protective in an hAPP mouse model. Although hAPP transgenic mice are considered a more chronic model of degeneration, as opposed to the KA lesion model, Aβ accumulation is sufficient for C-beclin to exacerbate neurodegenerative pathology.

### Beclin 1 cleavage sensitizes neurons to apoptosis

**Fig. 7:**
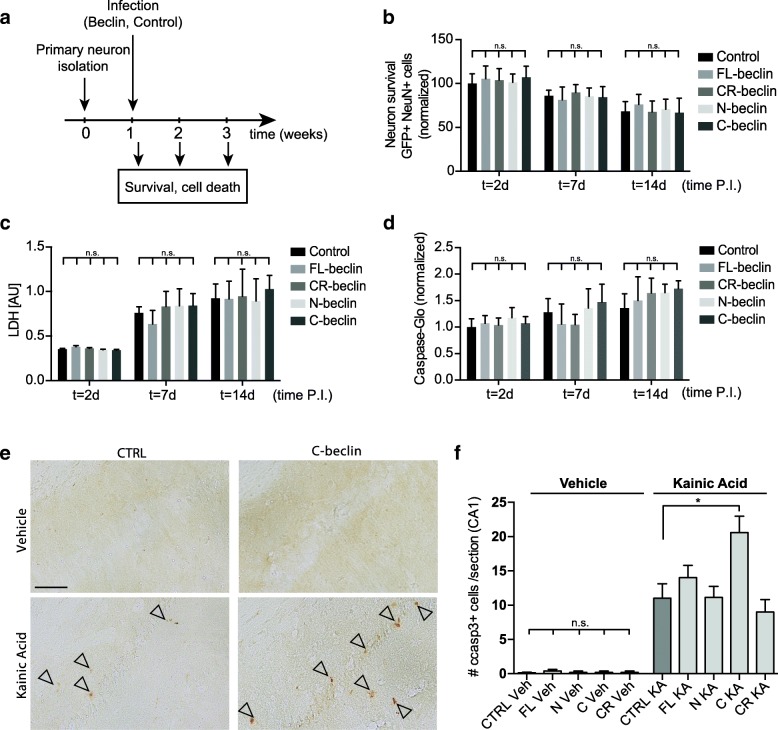
C-Beclin 1 expression potentiates caspase 3 activation in neurons. **a** Timeline of primary hippocampal neuron experiments. Hippocampal neurons were isolated from E16.5 embryos. Neurons were infected at 1 week in vitro with lentivirues expressing Beclin 1 and a fluorescent reporter from a neuron-specific Synapsin promoter. Survival and cell death was assessed 2, 7 and 14 days post infection (P.I.). **b** Quantification of neuronal survival up to 14 days post infection. The number of GFP-positive, NeuN-positive neurons is expressed relative to the 2d control group (*n* = 10 wells/group/time point, 5 fields/well). Data expressed as mean + SEM. **c** Quantification of lactate dehydrogenase (LDH) released into the media from damaged cells as an additional measure of cell death and survival (*n* = 10 wells/group/time point). Data expressed as mean + SD. **d** Baseline caspase activation and neuronal cell loss was further quantified on the basis of caspase3/7 activity using a luminescent caspase-Glo substrate assay (*n* = 10 /group/time point). Data expressed as mean + SD. Overexpression of full-length (FL) Beclin 1 or its caspase cleavage products does not impact neuronal survival and caspase activation in the absence of any neurodegenerative insults. **e** Caspase activation was assessed using cleaved-caspase3 specific immunostaining in the hippocampus of mice expressing Beclin 1 isoforms in combination with Kainic acid or vehicle treatment (scale bar 75 μm). **f** Quantification of cleaved caspase 3 neurons in the CA1 region (*n* = 5–6 mice/group for the vehicle condition, *n* = 8–20 mice/group for the Kainic acid condition; 4–5 sections per mouse brain). C-beclin expression exacerbates caspase 3 activation in response to excitotoxic insults in vivo. *p < 0.05; one-way ANOVA with a Tukey’s post test for multiple comparisons

To gain mechanistic insight into how Beclin 1 cleavage exacerbates neurodegeneration, we expressed the full-length, cleavage resistant, and cleavage fragments in primary hippocampal neurons (Fig. 7a and Additional file 9: Figure S9a). Neither the full-length nor the cleavage fragments lead to changes in neurite morphology (Additional file 9: Figure S9a-d). In line with the in vivo experiments (Additional file 4: Figure S4d-f), expression of Beclin 1 did not lead to any detectable changes in neuronal survival as assessed by neuronal cell counts and LDH release assays (Fig. 7b, c). Due to the well-established interaction of Beclin 1 with both apoptosis-associated proteins as well as regulation of autophagy, we next measured autophagy regulation in combination with Beclin 1 isoform expression. We measured autophagic flux in neuronal cell lines using a LC3-GFP reporter. As expected, overexpression of FL and CR-beclin increased the number of LC3-positive puncta [19, 20]. Importantly, expression of the two cleavage fragments did not alter autophagosome formation as compared to the control condition [29]. These findings were further corroborated in primary hippocampal neurons (Additional file 9: Figure S9e-h).

Previous studies established several connections between Beclin 1 and apoptosis. For example, Beclin 1 can directly interact with the anti-apoptotic protein Bcl-2, inhibiting the autophagic function of Beclin 1 [26, 27]. Additionally, previous studies in cell lines reported that C-beclin expression can trigger cytochrome c release from the mitochondria, a key step in the apoptotic process [28]. We therefore measured baseline caspase activation in primary neurons, but did not detect any increase in caspase activation between control and Beclin 1 overexpression conditions at any time point (Fig. 7d). We therefore hypothesized that Beclin 1 cleavage is not sufficient to induce neurodegeneration, but that it could sensitize neurons to potential stressors and insults. This notion is supported by our observation that Beclin 1 isoforms overexpression did not alter neuronal survival in vitro (Fig. 7b, c) or in vivo (Additional file 4: Figure S4d-g), but that C-beclin expression exacerbated neurodegeneration in the KA lesion model and in hAPP transgenic mice (Fig. 4c-f and Additional file 8: Figure S8). We therefore decided to treat neuronal cell lines expressing different Beclin 1 isoforms with Staurosporine. Using anti-cleaved caspase 3 specific immunolabeling, we detected a significant increase in caspase 3 activation in C-beclin expressing cells (Additional file 9: Figure S9i, j). Importantly, the FL-beclin and N-beclin conditions did not differ from the control group. Based on these in vitro findings, we next assessed caspase 3 activation in the KA lesion model in vivo. We did not detect any cleaved-caspase 3 immunostaining in the CA1 area of vehicle treated Beclin 1 overexpression animals. However, C-beclin overexpression significantly increase the number of caspase 3-positive cells in the KA lesion model (Fig. 7e, f).

Cumulatively, these findings demonstrate that C-beclin is not sufficient to trigger neuronal cell death, but can sensitize neurons to excitotoxic insults and exacerbate neurodegeneration in vitro and in vivo.

## Discussion

Our study provides several lines of evidence for the functional role of Beclin 1 cleavage in human disease and two mouse models of neurodegeneration. We demonstrate that Beclin 1 is cleaved in brain lysates of human AD patients. We utilized an acute neurodegenerative mouse model with neuronal cell loss in the CA1 region of the hippocampus, the brain area observed to have high Beclin 1 expression, and recapitulated the Beclin 1 cleavage phenotype observed in post-mortem brain tissue of human AD patients. Additionally, we detected a correlation between caspase 3 activation, the levels of Beclin 1 cleavage fragments and the degree of neurodegeneration and microglial activation in the hippocampus. Using a virus-mediated approach we demonstrated that overexpressing C-beclin exacerbates caspase 3 activation and neuronal cell loss in response to excitotoxic insults. Conversely, preventing proteolytic cleavage using a caspase inhibitor or a viral-mediated cleavage resistant Beclin 1 overexpression approach conferred neuroprotection. Using a hAPP transgenic mouse model of AD, we further demonstrated that expression of FL-beclin results in neuroprotection while expression of C-beclin exacerbates neurodegenerative phenotypes. These findings open the possibility to modulate the cleavage of Beclin 1 as a means by which to regulate neuronal cell death across neurodegenerative conditions.

Caspases play an important role in apoptotic cell death. Caspase activation has further been observed in AD and caspase inhibitor treatment has recently been shown to be beneficial in a mouse model of AD [58, 59]. As evidence for the involvement of caspases in neuronal cell death, we detected caspase activation in human AD brain samples and neuronal cell lines exposed to an apoptosis inducing drug. Moreover, caspase activation correlated with Beclin 1 cleavage in vitro and in vivo, suggesting that proteolytic cleavage of Beclin 1 plays a role in neurodegeneration. We sought to identify how the cleavage of FL-beclin, specifically the generation of the C-beclin fragment, contributes to the pathology of the KA lesion model of neurodegeneration. Although the extent to which caspase activation contributes to the pathology observed in this model is controversial [60–62], we were able to show contribution of apoptosis, and caspases in particular, to neuronal cell loss after injury. Furthermore, administration of caspase 3 inhibitor DEVD prior to KA lesion prevented Beclin 1 cleavage, showing that Beclin 1 is most likely a caspase substrate in vivo. Importantly, local DEVD administration in the hippocampus proved to be neuroprotective. It should be noted, however, that Beclin 1 is one of many potential caspase substrates that could mediate degeneration in the KA lesion model, with caspases having been shown to cleave various proteins including other autophagy-related proteins [31].

**Fig. 8:**
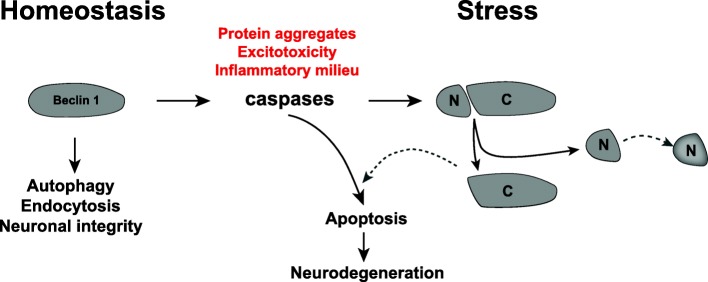
Beclin 1 as a switch between homeostasis and apoptosis in neurodegeneration. Schematic representation of the potential roles of Beclin 1. Under homeostatic conditions (left) Beclin 1 acts as a scaffold for different protein complexes mediating various cellular processes. Long-lived proteins and organelles can be degraded in the autophagy-lysosomal degradation pathway. Beclin 1 can also regulate endocytosis and receptor recycling, crucial processes for growth factor signaling in neurons. In response to stress (right) caspases become activated and cleave Beclin 1 into N-terminal and C-terminal fragments, impairing its interactions with other proteins and homeostatic functions. Additionally, the C-terminal fragment can prime the cell for apoptotic stimuli. In the context of neurodegeneration, the C-terminal fragment can potentiate the effect of caspase activation and exacerbate apoptosis and neuronal cell death

Through the use of a viral-mediated overexpression approach in conjunction with two neurodegenerative disease mouse models, we were able to tease apart the individual role of each Beclin 1 cleavage fragment in neurodegeneration with and without protein aggregation. We found that Beclin 1 can either exacerbate neurodegeneration or support the survival of neurons depending upon its cleavage state. While overexpressing FL-beclin has been previously shown to be beneficial in several models of neurodegeneration, such as hAPP overexpressing models of AD, α-synuclein models of PD and poly-glutamine disease models [5, 7, 18, 19], the beneficial effects observed thus far have been attributed to enhanced autophagy, increased clearance of aggregation-prone proteins and decreased amyloidosis and associated neuritic degeneration. Because our study utilized wild type animals, as well as hAPP transgenic models, we were able to examine the role of Beclin 1 both in the context of as well as independent of protein aggregation. Interestingly, it was selectively the caspase-resistant form of Beclin 1, but not FL-beclin, that was able to rescue neuronal cell loss in our excitotoxicity model of neurodegeneration. While at first these findings may appear disparate it should be noted that although FL-beclin can be overexpressed in our acute model of neurodegeneration it is still subject to caspase cleavage under stress [28, 29]. Therefore, increasing FL Beclin 1 continues to contribute to the production of its cleavage fragments exacerbating cell loss. In contrast, hAPP-based models are more chronic in nature and are not usually associated with acute cell lose. While our data indicate that Aβ accumulation is sufficient for C-beclin to exacerbate neurodegeneration, it may not prove strong enough to significantly deplete endogenous Beclin 1 level. In line with this, we previously did not observe a decrease in Beclin 1 levels in hAPP transgenic mice [5]. Therefore, overexpression of both the wild-type and caspase-resistant form of Beclin1 were sufficient to reduce Aβ pathology and associated degenerative phenotypes. Thus, the survival of neurons may be critically dependent on the nature and strength of the stressor and the levels of FL and cleaved Beclin 1 protein (Fig. 8). Subsequently, we believe that preventing the caspase cleavage of Beclin 1, and maintaining low levels of cleaved fragments, may prove to be of potential clinical interest.

In a broad context, we believe that the neuroprotective effect observed with the caspase-resistant form of Beclin 1 is due to two independent mechanisms. The first mechanism may prevent the stress-related deregulation of autophagy [63–65]. Beclin 1 cleavage may induce neurodegeneration by decreasing the FL-beclin protein, thereby mimicking the reduction observed in the Beclin 1-deficient mouse model and resulting in the autophagy associated neurodegenerative phenotypes [5, 12, 66]. Work in cell lines has also shown that caspase-cleavage of Beclin 1 leads to a loss of autophagic function, further substantiating this possibility. Furthermore, Beclin 1 cleavage fragments are not able to induce autophagy on their own, possibly because they cannot form functional complexes with other autophagy related proteins [28, 29]. However, they also do not appear to be acting as negative regulators of autophagy. Interestingly, autophagy has been reported to be up-regulated early on in excitotoxic and ischemic injuries, as a means to cope with the stress situation [4, 67]. Beclin 1 cleavage might therefore counteract this potentially beneficial effect of increased autophagy. Overexpressing a cleavage resistant form of Beclin 1 thereby assures that a constantly high level of the protein is present allowing it to fulfill its role in supporting the homeostatic energy balance of the cell (Fig. 8). Interestingly, a recently generated mouse model expressing a pro-autophagic form of FL-beclin with a single point mutation that disrupts the Bcl-2 Beclin 1 interaction, was demonstrated to increase health and lifespan in mice [68]. The same mouse was also shown to decrease Aβ plaque load and improve memory in mouse models of AD [69]. However, whether this model would confer protection in amyloidosis independent degenerative models such as the Kainic acid lesion models remains an open question.

We believe a second mechanism may generate a potentially pro-apoptotic C-terminal Beclin 1 fragment that results from Beclin 1 cleavage. We found that overexpressing C-beclin selectively in neurons in the CA1 region of the hippocampus exacerbated the neurodegeneration phenotype observed in the KA lesion model and hAPP transgenic mice. Surprisingly, no effect was observed in wildtype mice, suggesting that C-beclin only primes the cells to respond to stress stimuli but does not induce apoptosis itself. The same was observed in primary neurons where C-beclin expression did not alter caspase activation or neuronal survival. Additional studies in cell lines have shown that overexpressing the C-terminal fragment sensitizes cells for apoptosis. Furthermore, in a cell free system it was demonstrated that incubation of recombinant C-beclin with mitochondria leads to a release of proapoptotic factors such as cytochome C [28]. While recent studies suggest that N-beclin can also be detected in models of degeneration, thus far no function has been attributed to this cleavage product [32, 33]. Consistent with this, we did not observe any effect of N-terminal beclin expression on neuronal survival, autophagy or apoptosis. Interestingly, several other autophagy-related proteins have been shown to be cleaved during apoptosis [31], which is thought to be a way for cells to block cell survival and autophagy and commit cells towards an apoptotic cell fate. In particular, cleavage of Atg5, another autophagy related protein, has been shown to be able to induce apoptosis [70], suggesting that cleavage products of autophagy-proteins may be involved in the regulation of apoptosis. This body of research together with our in vivo study suggest that cleaved Beclin 1 may form a positive feedback loop on apoptosis and thereby exacerbate neurodegeneration in the presence of a stressor (Fig. 8). Hence, by overexpressing a cleavage resistant form of Beclin 1 the generation of the C-beclin fragment is reduced and in turn neuroprotection is conferred.

## Conclusions

Cumulatively, our data underscore the important role of apoptosis, caspase activation and Beclin 1 cleavage in neurodegeneration [3, 64, 65, 71]. Beclin 1 cleavage appears to be a crucial step in caspase-mediated apoptosis in neurons. Abrogating Beclin 1 cleavage enables neurons to survive after excitotoxic insult, caspase activation and in the context of amyloidosis. These data suggest that maintaining high levels of non-cleaved Beclin 1 is crucial for neuronal integrity and promoting neuronal survival during insults. Ultimately, our data indicate that Beclin 1 has versatile roles both in neuronal survival and apoptosis dependent upon its cleavage state. It is this duality of Beclin 1 function inherent in its cleavage state that positions Beclin 1 as an attractive candidate to be explored as a potential therapeutic target aimed at combating neuronal cell loss in a variety of neurodegenerative diseases by preventing its proteolytic cleavage.

## Additional files

Additional file 1: **Figure S1.** Beclin 1 expression in the mouse hippocampus. (PDF 4280 kb)

Additional file 2: **Figure S2.** Neurodegeneration and Beclin 1 cleavage in a Kainic acid-based mouse model. (PDF 5500 kb)

Additional file 3: **Figure S3.** Characterization of Beclin 1 AAV expression vectors. (PDF 399 kb)

Additional file 4: **Figure S4.** Adeno-associated viral vector-mediated spatial targeting of hippocampal CA1 neurons in vivo. (PDF 9270 kb)

Additional file 5: **Figure S5.** Characterization of Adeno-associated viral vector-mediated Beclin 1 overexpression in the hippocampus. (PDF 8890 kb)

Additional file 6: **Figure S6.** Stereotaxic Surgery is not sufficient to exacerbate neurodegeneration in the Kainic acid lesion model. (PDF 181 kb)

Additional file 7: **Figure S7.** Beclin 1 expression reduces Amyloid beta pathology in a hAPP mouse model. (PDF 2480 kb)

Additional file 8: **Figure S8.** C-Beclin expression exacerbates degeneration in a hAPP mouse model. (PDF 3420 kb)

Additional file 9: **Figure S9.** Beclin 1 expression impacts caspase 3 activation and autophagic flux in neuronal cells in vitro. (PDF 1050 kb)

## Abbreviations

AAV: Adeno-associated virus
AD: Alzheimer’s disease
Aβ: Amyloid beta
BH3: Bcl-2 homology-3 domain
C-beclin: C-terminal Beclin 1
CR-beclin: Caspase-resistant Beclin 1
DEVD: z-DEVD-FMK
FL-beclin: Full length Beclin 1
GFP: Green fluorescent protein
KA: Kainic acid
N-beclin: N-terminal Beclin 1
