# Supplementary material for: Proteolytic cleavage of Beclin 1 exacerbates neurodegeneration
Source: Mol Neurodegener. 2018 Dec 29;13:68. doi: 10.1186/s13024-018-0302-4 (PMC6310967; doi:10.1186/s13024-018-0302-4)
Supplement: Supplementary file 1 — Figure S1. Beclin 1 expression in the mouse hippocampus. (PDF 4280 kb) [file 13024_2018_302_MOESM1_ESM.pdf]

Supplementary Figure S1

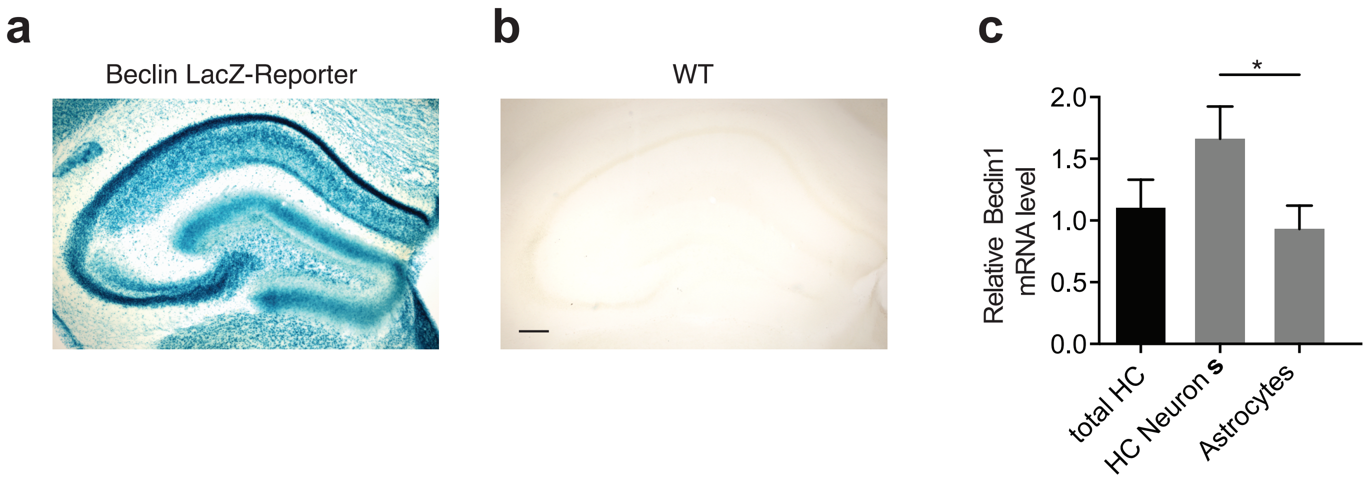

Supplementary Figure S1: Beclin 1 expression in the mouse hippocampus.

a,b β-Galactosidase activity assay was performed on Beclin 1 reporter mice expressing LacZ under the endogenous Beclin 1 locus. Representative hippocampal image of β-Galactosidase labeling of LacZ reporter (**a**) and WT littermate controls (**b**). β-Galactosidase expression was detected in the pyramidal cell layer in the hippocampal CA1 region of reporter mice but not WT mice (scale bar: 200μm) **c** Quantitative RT-PCR analysis of Beclin 1 expression in RNA isolated from hippocampi of adult 2 months old mice, primary hippocampal neuron and astrocyte cultures ($n = 5$ hippocampi, $n = 8$ for primary cultures). Data expressed as mean + SEM; $*p < 0.05$; compared by one-way ANOVA with a Tukey's post test for multiple comparisons.
