# Supplementary material for: Proteolytic cleavage of Beclin 1 exacerbates neurodegeneration
Source: Mol Neurodegener. 2018 Dec 29;13:68. doi: 10.1186/s13024-018-0302-4 (PMC6310967; doi:10.1186/s13024-018-0302-4)
Supplement: Supplementary file 2 — Figure S2. Neurodegeneration and Beclin 1 cleavage in a Kainic acid-based mouse model. (PDF 5500 kb) [file 13024_2018_302_MOESM2_ESM.pdf]

Supplementary Figure S2

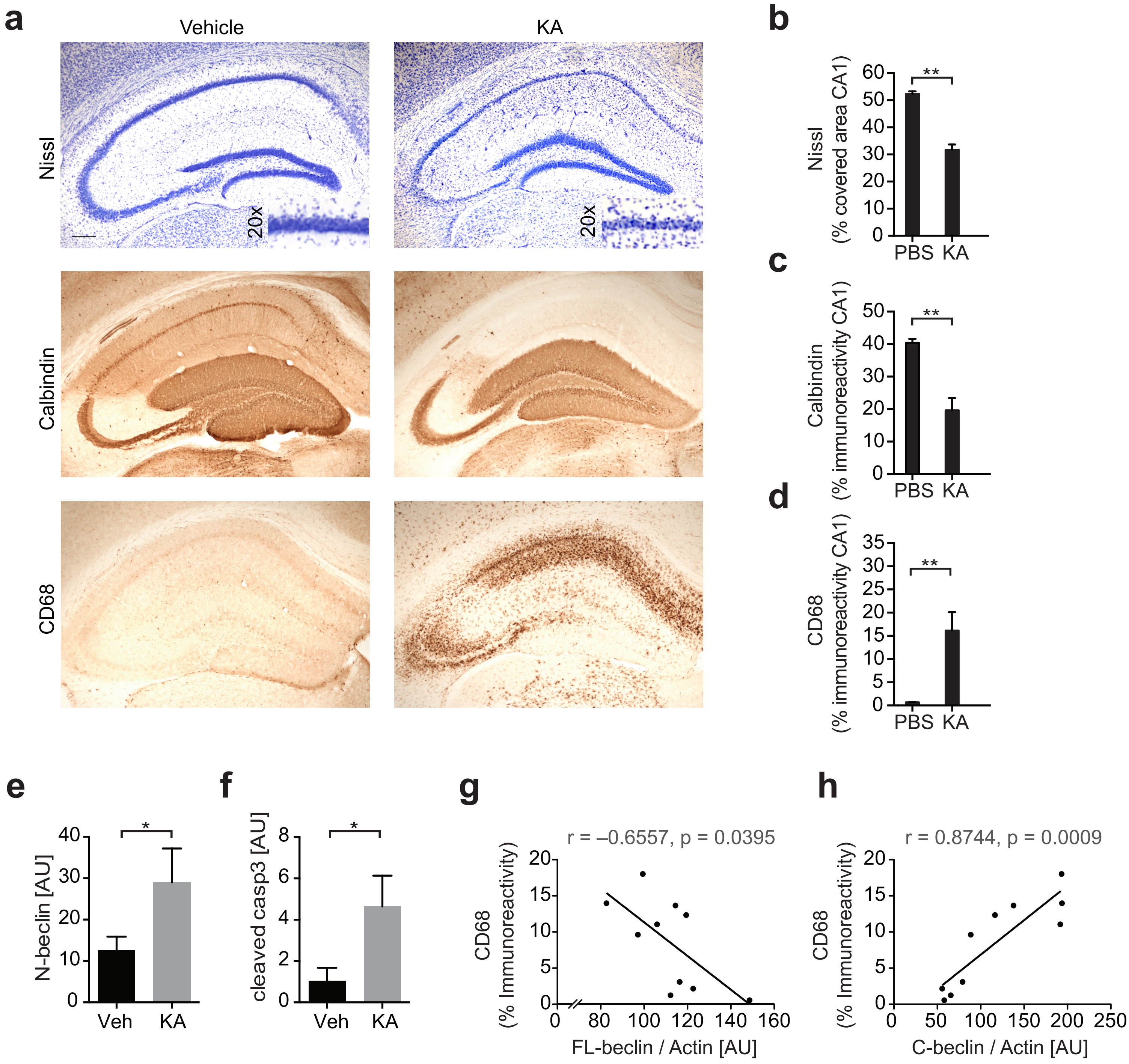

Supplementary Figure S2: Neurodegeneration and Beclin 1 cleavage in a Kainic acid-based mouse model.

a Neuronal injury after Kainic acid (KA) or vehicle treatment was assessed by Nissl staining (top panel) and immunohistochemical detection of the neuronal marker Calbindin (middle panel). Microglial activation was assessed by CD68 immunostaining (lower panel). Representative hippocampal images from adjacent sections of animals injected with Kainic acid (right side) or vehicle control (left side). Scale bar 200μm. **b-d** Quantification of Nissl staining (**b**), Calbindin immunostaining (**c**) and CD68 immunostaining (**d**) expressed as percentage area covered by staining in the CA1 region of the hippocampus (n =10 mice; 3-4 sections per mouse brain). **e**,**f** Quantification of N-beclin and cleaved-caspase 3 level in hippocampal lysates of Kainic acid and vehicle treated animals using Western blot analysis (n = 10/group). **g,h** Correlation of FL-Beclin 1 level (**g**) and C-Beclin 1 level (**h**) with the microglial activation marker CD68. Beclin 1 was assessed by Western blot, CD68 was measured by immunohistochemistry. Data expressed as mean + SEM; *$p < 0.05$; **$p < 0.01$; compared by unpaired Student's t-test. Pearson's correlation.
