# Supplementary material for: Proteolytic cleavage of Beclin 1 exacerbates neurodegeneration
Source: Mol Neurodegener. 2018 Dec 29;13:68. doi: 10.1186/s13024-018-0302-4 (PMC6310967; doi:10.1186/s13024-018-0302-4)
Supplement: Supplementary file 3 — Figure S3. Characterization of Beclin 1 AAV expression vectors. (PDF 399 kb) [file 13024_2018_302_MOESM3_ESM.pdf]

Supplementary Figure S3

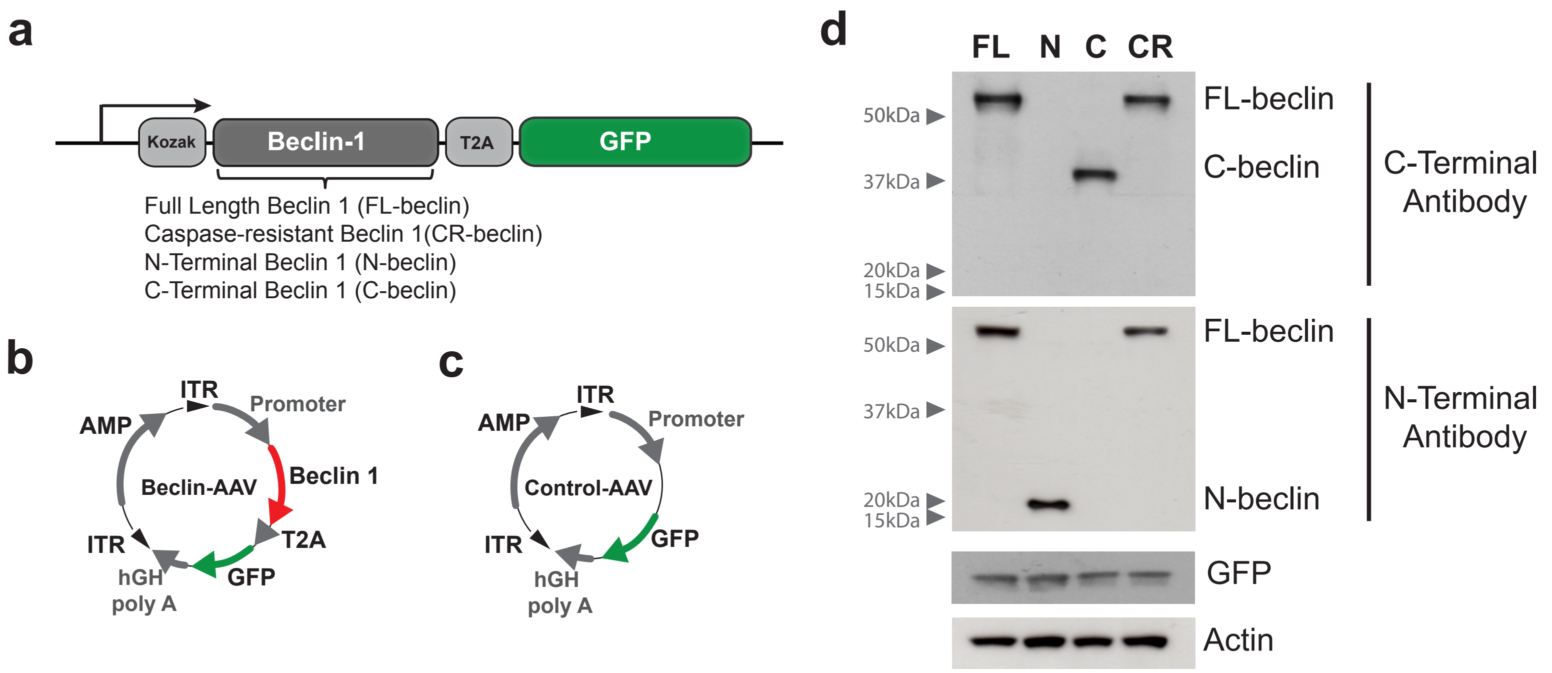

Supplementary Figure S3: Characterization of Beclin 1 AAV expression vectors.

a Schematic of bicistronic Beclin 1 and GFP reporter cassette. Beclin 1 is expressed in frame with a self-cleaving T2a and GFP. Four plasmids expressing different Beclin 1 constructs (FL, N, C, CR) were generated. **b,c** Schematic representation of adeno-associated viral (AAV) Beclin 1 and control plasmids. Abbreviations: AMP, ampicillin; ITR, inverted terminal repeats; GFP, green fluorescent protein; hGH poly A, human growth hormon polyA sequence. **d** Characterization of Beclin 1 plasmids and N-terminal and C-terminal specific antibodies in Neuro-2a neuroblastoma cell line. Representative Western blot of neuronal cells transduced with Beclin 1 constructs, probed with anti-C-terminal Beclin 1, anti-N-terminal Beclin 1, anti-hrGFP and anti-Actin antibodies. Arrowheads indicate molecular weight markers (in kDa). Both terminal-specific antibodies recognize the respective fragment at the expected molecular weight as well as the full length (FL) form.
