# Supplementary material for: Proteolytic cleavage of Beclin 1 exacerbates neurodegeneration
Source: Mol Neurodegener. 2018 Dec 29;13:68. doi: 10.1186/s13024-018-0302-4 (PMC6310967; doi:10.1186/s13024-018-0302-4)
Supplement: Supplementary file 4 — Figure S4. Adeno-associated viral vector-mediated spatial targeting of hippocampal CA1 neurons in vivo. (PDF 9270 kb) [file 13024_2018_302_MOESM4_ESM.pdf]

Supplementary Figure S4

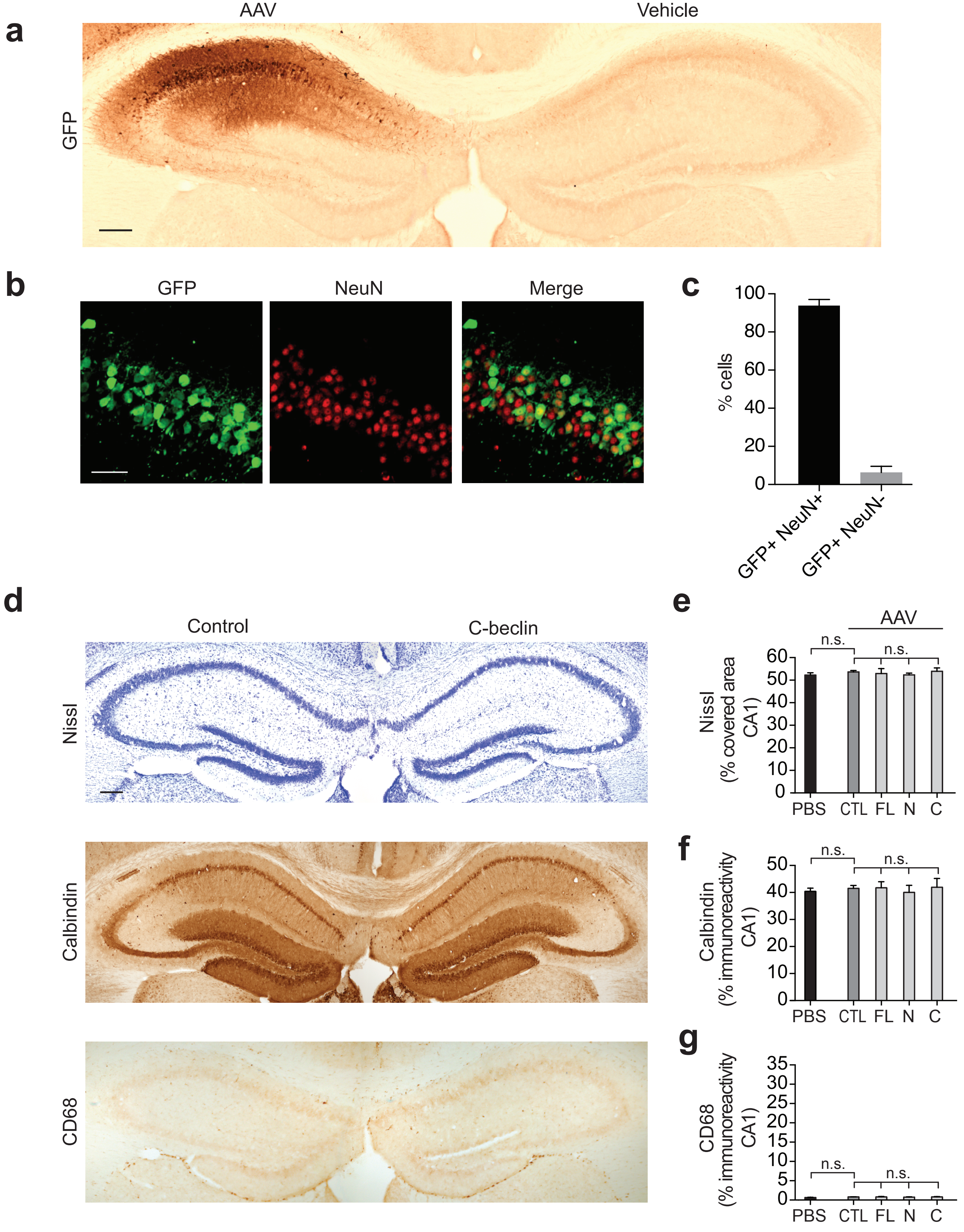

Supplementary Figure S4: Adeno-associated viral vector-mediated spatial targeting of hippocampal CA1 neurons *in vivo*.

a Characterization of AAV expression *in vivo*. High titer Beclin 1-AAV vectors were used to overexpress Beclin 1 and GFP in hippocampal neurons of adult mice. Representative image of immunostaining against GFP following unilateral stereotaxic delivery of control AAV into the CA1 region of the hippocampus (scale bar: 200μm). Strong ipsilateral labeling can be observed in the pyramidal cell body layer and neurites three weeks post infection. **b** Brain sections were double-immunolabeled with antibodies against GFP and neuronal cell type specific marker NeuN. Images were taken by confocal microscopy (scale bar: 50μm). **c** Quantification of GFP-positive and NeuN-positive cells (n = 5 mice, 3-4 hippocampal sections/mouse). **d** Adult mice were stereotaxically injected with Beclin 1-AAV into one hemisphere and Control-AAV into the contralateral side. Animals were sacrificed 3- 4 weeks after surgery and brains were analyzed. Neuronal integrity after viral overexpression of Beclin 1 or control was assessed by Nissl staining (top panel) and immunohistochemical detection of the neuronal marker Calbindin (middle panel). Microglial activation was assessed by CD68 immunostaining (lower panel). Representative hippocampal images from adjacent sections of one animal expressing C-terminal Beclin 1 (right side) and Control (left) are shown (scale bar: 200μm). **e-g** Quantification of Nissl staining (**e**), Calbindin immunostaining (**f**) and CD68 immunostaining (**g**) expressed as percentage area covered by staining in the CA1 region of the hippocampus. Control (CTL), Full length (FL), N-terminal (N) and C-terminal (C) beclin groups were quantified and compared to animals (PBS group) that did not undergo stereotaxic surgery (n = 10 mice/group for uninjected group; n = 5-6 mice/group for Beclin and Control expression conditions; 4-5 hippocampal sections/mouse brain). Data expressed as mean

+ SEM, compared by unpaired Student's t-test or one-way ANOVA with a Tukey's post test for multiple comparisons.
