# Supplementary material for: Proteolytic cleavage of Beclin 1 exacerbates neurodegeneration
Source: Mol Neurodegener. 2018 Dec 29;13:68. doi: 10.1186/s13024-018-0302-4 (PMC6310967; doi:10.1186/s13024-018-0302-4)
Supplement: Supplementary file 5 — Figure S5. Characterization of Adeno-associated viral vector-mediated Beclin 1 overexpression in the hippocampus. (PDF 8890 kb) [file 13024_2018_302_MOESM5_ESM.pdf]

Supplementary Figure S5

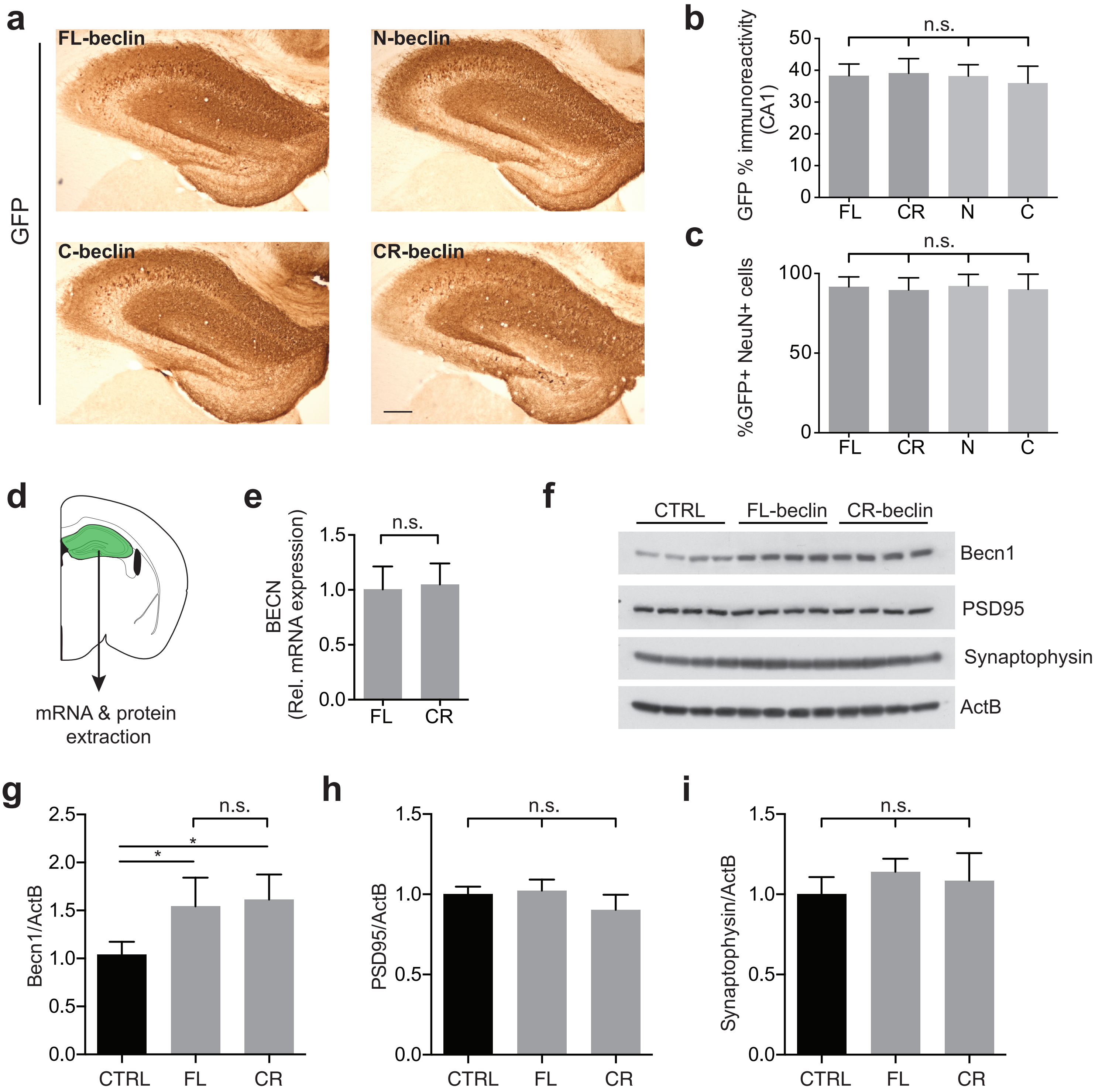

Supplementary Figure S5: Characterization of Adeno-associated viral vector-mediated Beclin 1 overexpression in the hippocampus.

a Characterization of GFP expression in Beclin 1-T2a-GFP AAV injected animals. Representative hippocampal images of GFP labeling. Scale bar 200μm. **b** Quantification of GFP immunostaining in the CA1 region of the hippocampus. Full length (FL), caspase-resistant (CR), N-terminal (N) and C-terminal (C) beclin groups were quantified (n = 5 mice/group; 3-4 hippocampal sections/mouse brain). **c** Quantification of GFP-positive and NeuN-positive cells (n = 5 mice/group, 3-4 hippocampal sections/mouse). **d** Schematic of experimental design. Hippocampi from FL-beclin, CR-beclin and control AAV injected mice were extracted and used for mRNA and protein expression analysis. **e** qRT-PCR analysis of BECN1 mRNA expression in FL-beclin and CR-beclin overexpressing hippocampi. **f-i** Representative Western blot (**f**) and quantification of hippocampal lysates from FL-beclin, CR-beclin and control AAV expressing mice, probed with anti-Becn1 (**g**), anti PSD95 (**h**), anti Synaptophysin (**i**) and anti-Actin (ActB) antibodies. Actin was used as a loading control for normalization (n=5/group). Data expressed as mean + SEM, compared by unpaired Student's t-test or one-way ANOVA with a Tukey's post test for multiple comparisons.
