# Supplementary material for: Proteolytic cleavage of Beclin 1 exacerbates neurodegeneration
Source: Mol Neurodegener. 2018 Dec 29;13:68. doi: 10.1186/s13024-018-0302-4 (PMC6310967; doi:10.1186/s13024-018-0302-4)
Supplement: Supplementary file 6 — Figure S6. Stereotaxic Surgery is not sufficient to exacerbate neurodegeneration in the Kainic acid lesion model. (PDF 181 kb) [file 13024_2018_302_MOESM6_ESM.pdf]

Supplementary Figure S6

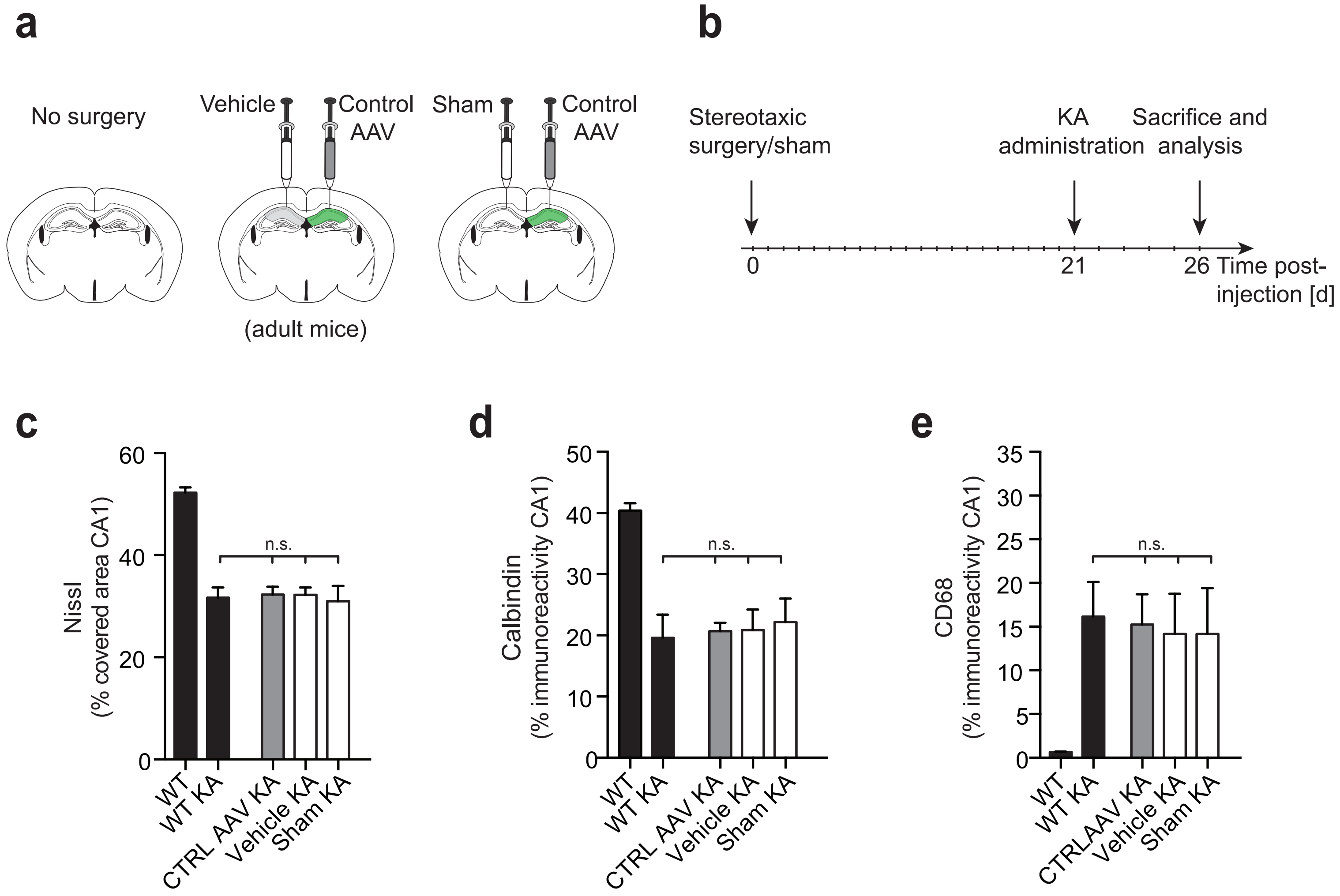

Supplementary Figure S6: Stereotaxic Surgery is not sufficient to exacerbate neurodegeneration in the Kainic acid lesion model.

a Schematic of experimental groups. Adult mice were stereotaxically injected into the CA1 region of the hippocampus with control AAV, Vehicle (PBS) or underwent a sham injection without the delivery of any solution. A wildtype group (with no surgery) was included as well. **b** Experimental time line of stereotaxic surgery followed by Kainic acid (KA) or PBS administration in adult mice. **c-e** Neuronal integrity was assessed by Nissl staining (**c**) and immunohistochemical detection of the neuronal marker Calbindin (**d**) and microglial activation was assessed by CD68 immunostaining (**e**). Quantifications expressed as percentage area covered by staining in the CA1 region of the hippocampus (n = 8-12 mice/group, 4-5 hippocampal sections/brain). Data expressed as mean + SEM, compared by one-way ANOVA with a Tukey's post test for multiple comparisons.
