# Supplementary material for: Proteolytic cleavage of Beclin 1 exacerbates neurodegeneration
Source: Mol Neurodegener. 2018 Dec 29;13:68. doi: 10.1186/s13024-018-0302-4 (PMC6310967; doi:10.1186/s13024-018-0302-4)
Supplement: Supplementary file 7 — Figure S7. Beclin 1 expression reduces Amyloid beta pathology in a hAPP mouse model. (PDF 2480 kb) [file 13024_2018_302_MOESM7_ESM.pdf]

Supplementary Figure S7

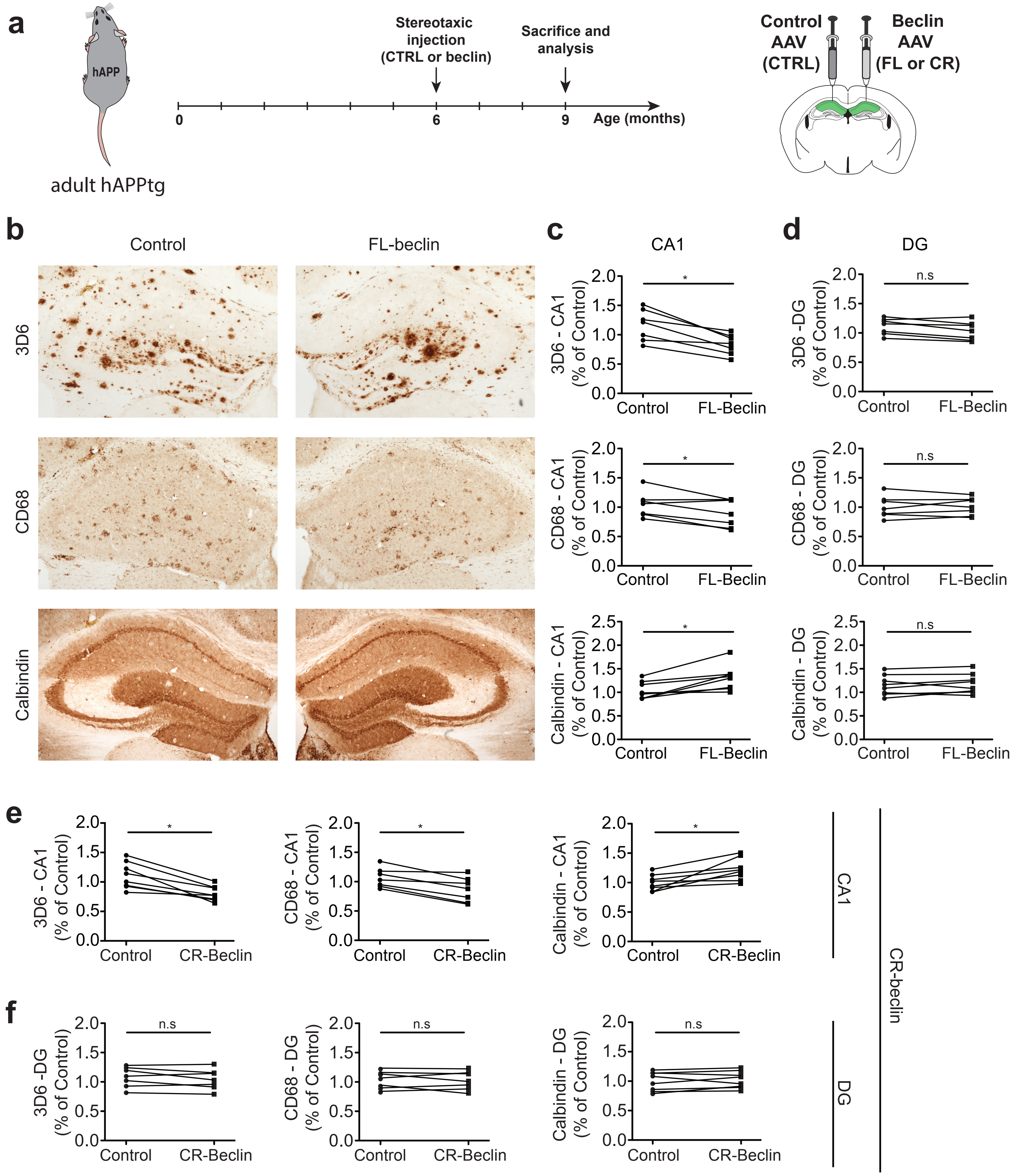

Supplementary Figure S7: Beclin 1 expression reduces Amyloid beta pathology in a hAPP mouse model.

a Schematic of experimental design and time line. 6 months old hAPP-transgenic mice were stereotaxically injected with FL-beclin or CR-beclin into the CA1 and Control AAV into the contralateral hemisphere. Dentate gyrus (DG) was used as a control area not targeted by the AAV injections. Mice were analyzed after 3 months. **b** Amyloid beta (Aβ) pathology and associated degenerative phenotypes were assessed by immunohistochemical detection of Aβ (using the 3D6 antibody (top panel), microglial activation marker CD68 (middle panel) and neuronal marker Calbindin (lower panel). Representative hippocampal images from adjacent sections of one animal expressing FL-beclin (right hemisphere) or control (left hemisphere). Scale bar 200μm. **c,d** Quantification of 3D6, CD68 and Calbindin immunostaining in the CA1 (**c**) and dentate gyrus area (**d**) of the hippocampus comparing the FL-beclin expressing to the contralateral control hemispheres. Beclin 1 and control AAV were selectively expressed in the CA1 area. **e,f** Quantification of 3D6, CD68 and Calbindin immunostaining in the CA1 (**e**) and dentate gyrus area (**f**) of the hippocampus in CR-beclin and control injected animals (n = 7 mice/group; 4-5 hippocampal sections/brain). Data expressed as mean value for each hemisphere of the same animal; *$p < 0.05$; compared by paired Student's t-test.
