# Supplementary material for: Proteolytic cleavage of Beclin 1 exacerbates neurodegeneration
Source: Mol Neurodegener. 2018 Dec 29;13:68. doi: 10.1186/s13024-018-0302-4 (PMC6310967; doi:10.1186/s13024-018-0302-4)
Supplement: Supplementary file 8 — Figure S8. C-Beclin expression exacerbates degeneration in a hAPP mouse model. (PDF 3420 kb) [file 13024_2018_302_MOESM8_ESM.pdf]

Supplementary Figure S8

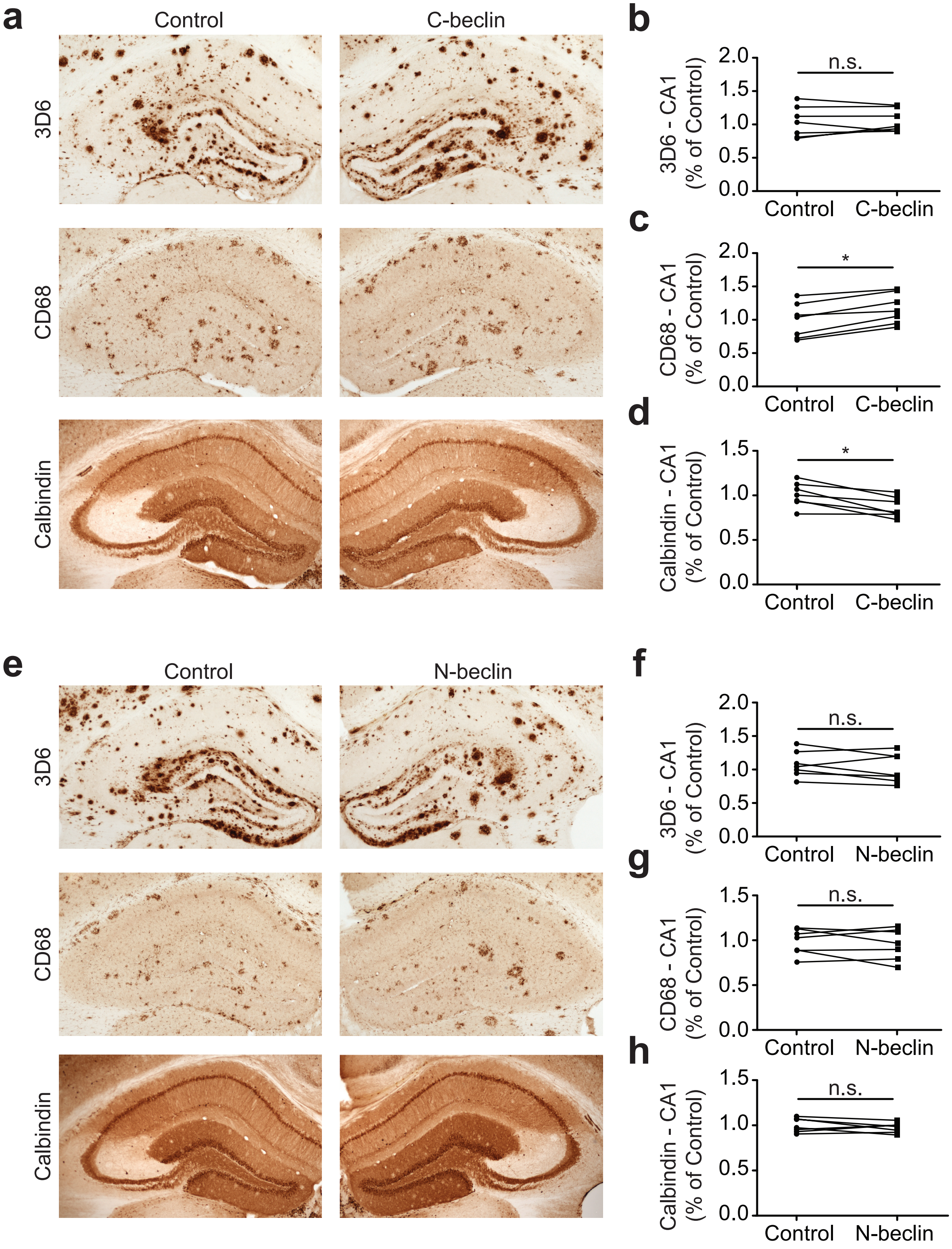

Supplementary Figure S8: C-Beclin expression exacerbates degeneration in a hAPP mouse model.

a 6 months old hAPP-transgenic mice were stereotaxically injected with C-beclin into the CA1 of the hippocampus and control AAV at the same site into the contralateral hemisphere. Amyloid beta (Aβ) pathology and associated degenerative phenotypes were assessed by immunohistochemical detection of Aβ (using the 3D6 antibody (top panel), microglial activation marker CD68 (middle panel) and neuronal marker Calbindin (lower panel). Representative hippocampal images from adjacent sections of one animal expressing C-beclin fragments (right hemisphere) or control (left hemisphere). Scale bar 200μm. **b-d** Quantification of 3D6 (**b**), CD68 (**c**) and Calbindin (**d**) immunostaining in the CA1 area of the hippocampus. Comparison of C-beclin to the contralateral control hemisphere (n=7 mice/group; 4-5 hippocampal sections/brain). **e** 6 months old hAPP-transgenic mice were stereotaxically injected with N-beclin into the CA1 of the hippocampus and control AAV at the same site into the contralateral hemisphere. Amyloid beta (Aβ) pathology and associated degenerative phenotypes were assessed by immunohistochemical detection of Aβ (using the 3D6 antibody (top panel), microglial activation marker CD68 (middle panel) and neuronal marker Calbindin (lower panel). Representative hippocampal images from adjacent sections of one animal expressing N-beclin fragments (right hemisphere) or control (left hemisphere). Scale bar 200μm. **f-h** Quantification of 3D6 (**f**), CD68 (**g**) and Calbindin (**h**) immunostaining in the CA1 area of the hippocampus. Comparison of N-beclin to the contralateral control hemisphere (n=7 mice/group; 4-5 hippocampal sections/brain). Data expressed as mean value for each hemisphere of the same animal; $*p < 0.05$; compared by paired Student's t-test.
