# Supplementary material for: Proteolytic cleavage of Beclin 1 exacerbates neurodegeneration
Source: Mol Neurodegener. 2018 Dec 29;13:68. doi: 10.1186/s13024-018-0302-4 (PMC6310967; doi:10.1186/s13024-018-0302-4)
Supplement: Supplementary file 9 — Figure S9. Beclin 1 expression impacts caspase 3 activation and autophagic flux in neuronal cells in vitro. (PDF 1050 kb) [file 13024_2018_302_MOESM9_ESM.pdf]

Supplementary Figure S9

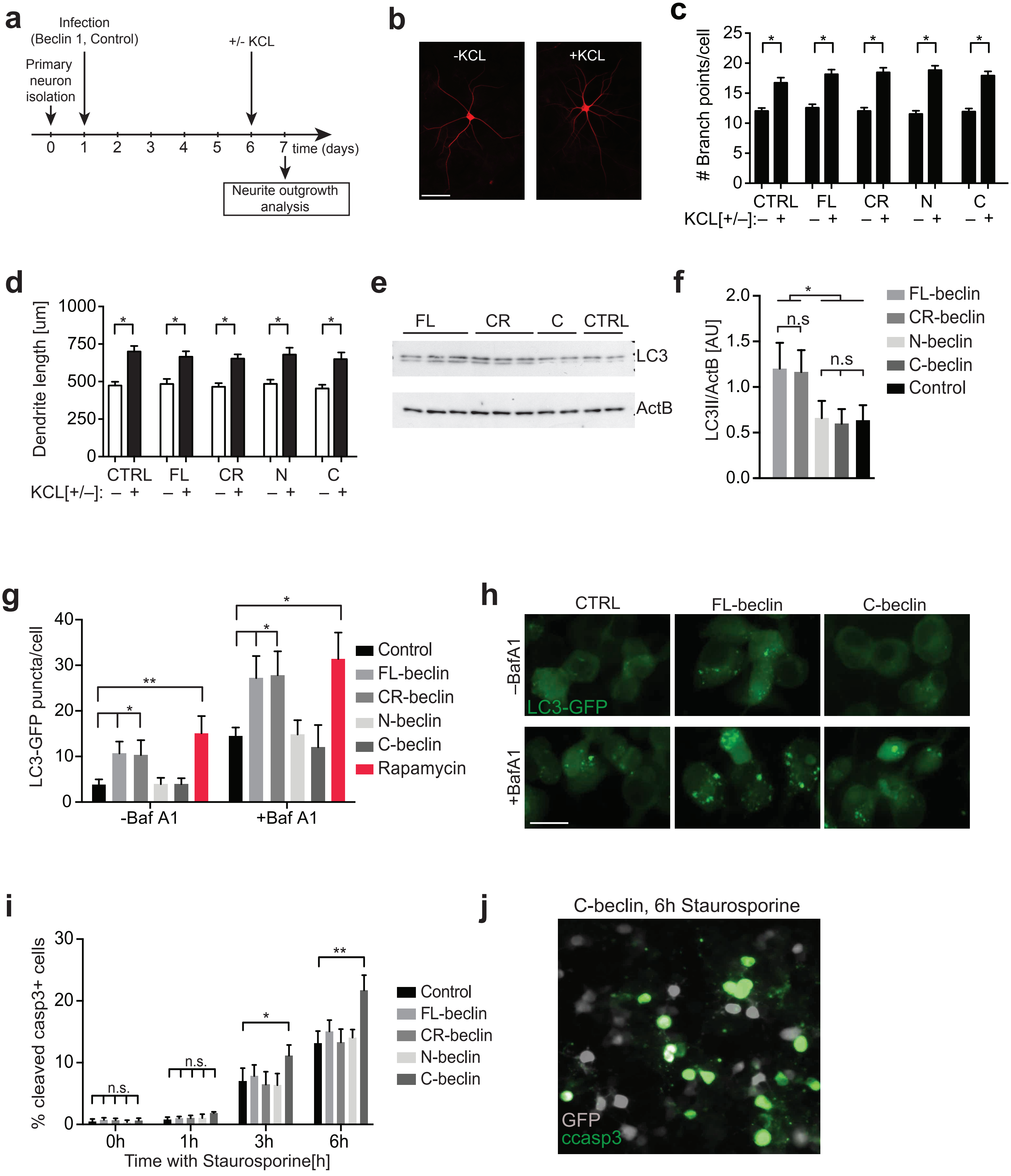

Supplementary Figure S9: Beclin 1 expression impacts caspase 3 activation and autophagic flux in neuronal cells *in vitro*.

a Timeline of primary hippocampal neuron experiments. Hippocampal neurons were isolated from E16.5 embryos. Neurons were infected at 1 day *in vitro* with lentivirues expressing Beclin 1 and a fluorescent reporter from a neuron-specific Synapsin promoter. Neurons were temporarily depolarized with 30mM KCL prior to neurite outgrowth analysis at 6 days in culture. **b** Neurons were labeled with antibodies against the marker MAP2. Examples of primary neurons with or withought KCL depolarization (scale bar: 30uM). **c,d** Quantification of the number of branch points and total dendrite length. Beclin 1 overexpression did not alter neurite outgrowth in primary hippocampal neurons in response to KCL depolarization. 10–15 neurons were scored per coverslip with 4 coverslips/condition. **e,f** Representative Western blot (**e**) and quantification (**f**) of primary neurons expressing different Beclin 1 forms, probed with anti-LC3 and anti-Actin (ActB) antibodies (n=4/group). Neurons were infected at 7 days *in vitro* and LC3 level were analyzed at 14 days *in vitro*. **g,h** LC3-GFP Neuro-2a reporter cell lines were transduced with different Beclin 1 constructs. GFP-positive puncta were counted using MetaExpress software (**g**). Rapamycin was used as known inducer of autophagy. Autophagic flux was measured after treatment with bafilomycin A (Baf A1), an inhibitor of lysosomal acidification, preventing autophagosome–lysosomal fusion. Overexpression of full length Beclin 1 forms (FL and CR), but not the cleavage fragments (N or C), increases the number of LC3-GFP puncta. **i** Neuro-2a cell lines stably expressing full length (FL or CR) Beclin 1 or its cleavage fragments (N or C) were exposed to 1μM Staurosporine for up to 6 hours. Cleaved caspase 3 was used as a marker for caspase activation. C-beclin expression exacerbates caspase 3 activation in response to Staurosporine treatment. **j** Example of activated cleaved caspase 3 in Staurosporine-treated C-beclin overexpressing neuronal cells *in vitro* (green:

cleaved caspase 3, scale bar: 30uM). Data expressed as mean + SEM; *p < 0.05; **p < 0.01 compared by unpaired Student's t-test or one-way ANOVA with a Tukey's post test for multiple comparisons.
